# Spatiotemporal abnormality dynamics of the pale grass blue butterfly: three years of monitoring (2011–2013) after the Fukushima nuclear accident

**DOI:** 10.1186/s12862-015-0297-1

**Published:** 2015-02-10

**Authors:** Atsuki Hiyama, Wataru Taira, Chiyo Nohara, Mayo Iwasaki, Seira Kinjo, Masaki Iwata, Joji M Otaki

**Affiliations:** The BCPH Unit of Molecular Physiology, Department of Chemistry, Biology and Marine Science, University of the Ryukyus, Okinawa, 903-0213 Japan

**Keywords:** Adaptive evolution, Fukushima nuclear accident, Long-term monitoring, Pale grass blue butterfly, Radioactive contamination

## Abstract

**Background:**

Long-term monitoring of the biological impacts of the radioactive pollution caused by the Fukushima nuclear accident in March 2011 is required to understand what has occurred in organisms living in the polluted areas. Here, we investigated spatial and temporal changes of the abnormality rate (AR) in both field-caught adult populations and laboratory-reared offspring populations of the pale grass blue butterfly, *Zizeeria maha*, which has generation time of approximately one month. We monitored 7 localities (Fukushima, Motomiya, Hirono, Iwaki, Takahagi, Mito, and Tsukuba) every spring and fall over 3 years (2011–2013).

**Results:**

The adult ARs of these localities quickly increased and peaked in the fall of 2011, which was not observed in non-contaminated localities. In the offspring generation, the total ARs, which include deaths at the larval, prepupal, and pupal stages and morphological abnormalities at the adult stage, peaked either in the fall of 2011 or in the spring of 2012, with much higher levels than those of the parent field populations, suggesting that high incidence of deaths and abnormalities might have occurred in the field populations. Importantly, the elevated ARs of the field and offspring populations settled back to a normal level by the fall of 2012 and by the spring of 2013, respectively. Similar results were obtained not only in the spatiotemporal dynamics of the number of individuals caught per minute but also in the temporal dynamics of the correlation coefficient between the adult abnormality rate and the ground radiation dose or the distance from the Power Plant.

**Conclusions:**

These results demonstrated an occurrence and an accumulation of adverse physiological and genetic effects in early generations, followed by their decrease and leveling off at a normal level, providing the most comprehensive record of biological dynamics after a nuclear accident available today. This study also indicates the importance of considering generation time and adaptive evolution in evaluating the biological impacts of artificial pollution in wild organisms.

**Electronic supplementary material:**

The online version of this article (doi:10.1186/s12862-015-0297-1) contains supplementary material, which is available to authorized users.

## Background

The release of a massive amount of radioactive materials from the Fukushima Dai-ichi nuclear power plant (FNPP) to the surrounding environment on 15 March 2011 and afterwards resulted in large-scale radioactive pollution worldwide and especially severe pollution in the Tohoku and Kanto districts of Japan [1,2]. Both marine and forest ecosystems have been heavily polluted [3,4], but scientific studies on biological impacts of this accident are still scarce. Yet, such studies are gradually accumulating now, which includes changes in abundance of animals, especially birds and butterflies, in the polluted areas [5,6]. Recently, low blood cell counts have been reported in wild Japanese monkeys [7]. In gall-forming aphids, severe morphological abnormalities have been documented from Fukushima samples, which are rare from other samples [8]. In some of these studies, insects played an important role as environmental indicators.

Coincidentally, another insect, the pale grass blue butterfly, *Zizeeria maha*, has been used to examine the biological effects of the accident [9-14]. This small butterfly has many advantages over other animals as an environmental indicator [12-14]. It is a multivoltine insect, having a life cycle of about a month from May to November in the Tohoku and Kanto districts. Because larvae of this butterfly cannot grow rapidly in winter, approximately six or seven generations occur per year in the Tohoku and Kanto districts. This butterfly is completely dependent on a single host plant, *Oxalis corniculata*. Partly because this plant is very small in height, this butterfly species lives on or near the surface of the ground throughout its life stages. It thus responds strongly to changes in the ground surface environment. Additionally, methods for its rearing in a small laboratory space have been well developed [15].

In this butterfly, morphological abnormalities and reduced wing size have been documented in field-caught adult samples from the polluted areas in 2011 [9]. Moreover, these observations in the field populations have been supported by offspring (F_1_) rearing experiments in the laboratory, resulting in a high incidence of deaths, morphological abnormalities, and growth retardation [9]. Furthermore, external and internal irradiation experiments have reproduced the field observations, including the high mortality rate, high morphological abnormality rate, and reduced wing size, strongly suggesting the causality of the pollution [9]. Also in that study, morphological abnormalities detected in the spring and fall of 2011 were compared, showing a possible accumulation of genetic and physiological damage in the fall of 2011 [9]. The heritability of abnormal traits by the offspring generations was also demonstrated [9]. However, long-term monitoring of the field populations is critical to correctly understand the impacts on pale grass blue butterfly populations in polluted areas.

Following the high mortality and abnormality in 2011 [9], 3 scenarios may be hypothesized. First, the high mortality and abnormality are kept for the following years (extended mortality hypothesis). Second, the populations experience even higher mortality and abnormality, eventually resulting in extinction (extinction hypothesis). Third, the populations gradually gain normality (normalization hypothesis). Temporal data were required to test these hypotheses.

**Figure 1:**
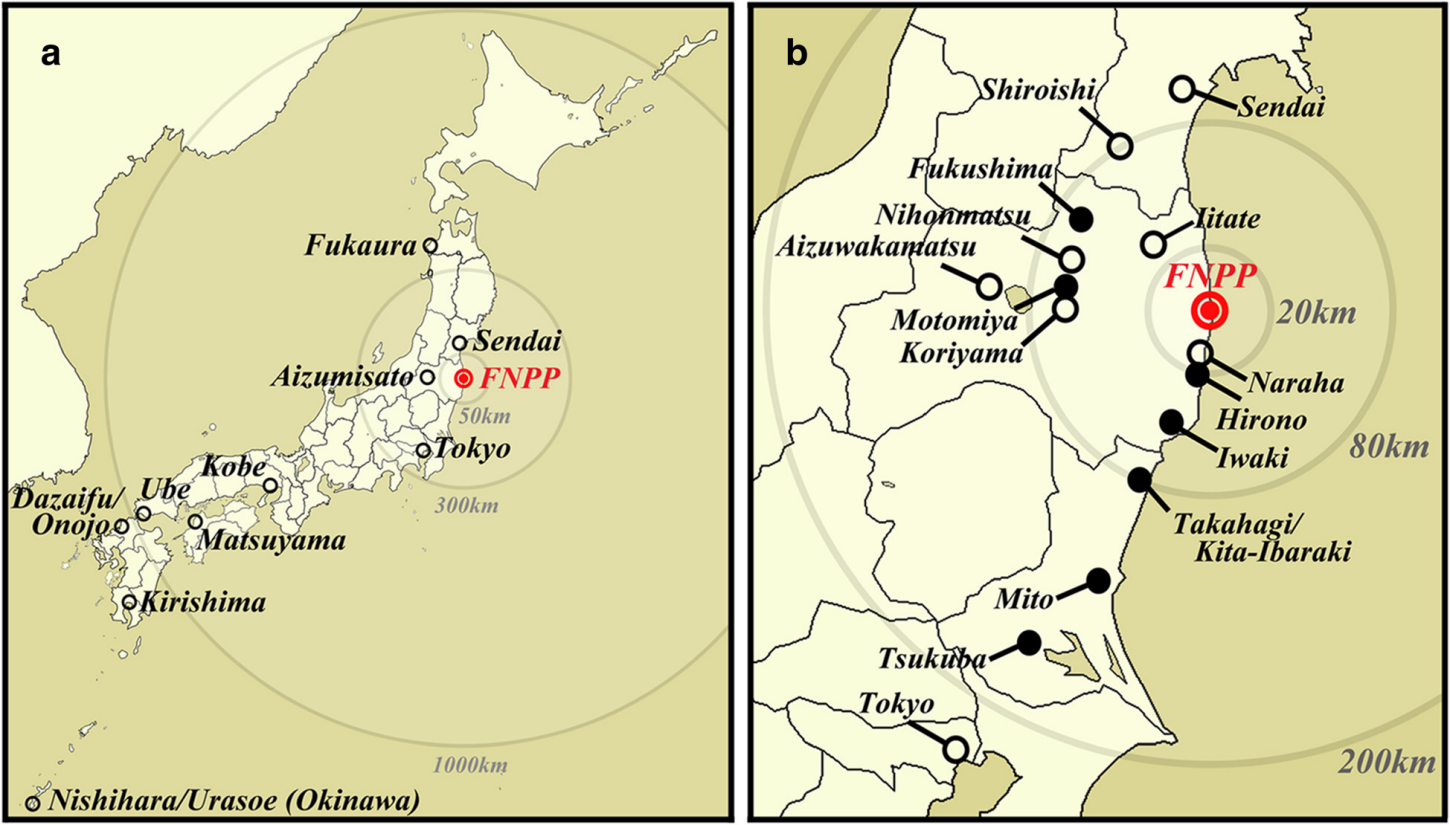
**Collection localities for the adult butterfly samples. (a)** A map of Japan. Minor collection localities for control samples are shown together with the Fukushima Dai-ichi Nuclear Power Plant (FNPP) in red. Distances from the FNPP are indicated by circles. **(b)** A map of the Tohoku and Kanto districts. The seven major localities from which the adult samples were collected are shown by black dots. Takahagi and Kita-Ibaraki are treated as a single locality, “Takahagi”, in this study because of their proximity. Minor localities are shown by open circles. The FNPP is shown in red. Distances from the FNPP are indicated by circles.

In the present study, we present our 3-year monitoring results of the pale grass blue butterfly in the Tohoku and Kanto districts from 2011 to 2013. We performed field surveys in the spring and fall every year, mainly visiting the following 7 localities: Fukushima, Motomiya, Hirono, Iwaki, Takahagi (including Kita-Ibaraki throughout this study), Mito, and Tsukuba (Figure 1; Additional file 1: Tables S1-S6). We then examined the morphological abnormalities of the collected adults from the field populations (defined as the parent or P generation), expressed as the adult abnormality rate (aAR). In addition, we obtained the offspring generation (defined as the offspring or F_1_ generation) from the collected adults, and we obtained the total abnormality rate (tAR), which includes deaths at the larval, prepupal, and pupal stages and the morphological abnormalities of the surviving adults. The offspring observations provided us with invaluable information on the field populations, including the heritability of abnormalities, and on a natural selection process that has occurred in the polluted environment.

## Results

### The dynamics of the ground radiation dose

**Figure 2:**
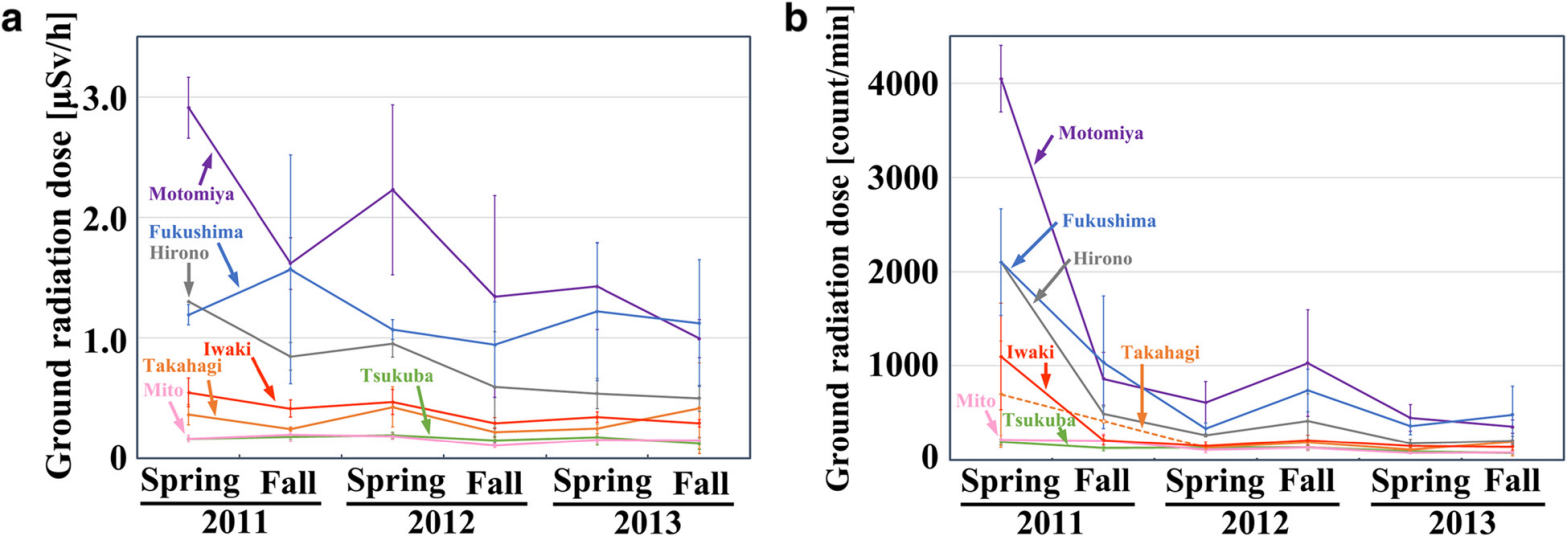
**Dynamics of the radioactivity levels on the ground of the 7 major collection localities in 2011–2013. (a)** Radioactivity measured with a scintillation counter [μSv/h]. **(b)** Radioactivity measured with a GM counter [count/min]. Error bars indicate the mean ± SE. Data point of Takahagi in the fall of 2011 was not obtained.

The ground radiation dose at the collection sites in the 7 localities was measured at the time of butterfly sample collection in 6 sampling attempts over 3 years. The ground radiation dose reached its maximum level in the spring of 2011 and declined afterwards (Figure 2a, b), likely due to fast-decaying radionuclides. However, even in the fall of 2013, considerable levels were detected, especially from Fukushima and Motomiya. The level of Hirono was also high. It is important to note that the ground radiation doses in these localities in the fall of 2013 were still higher than the doses in other localities in the spring of 2011.

### The aAR dynamics of the field-collected adults

**Figure 3:**
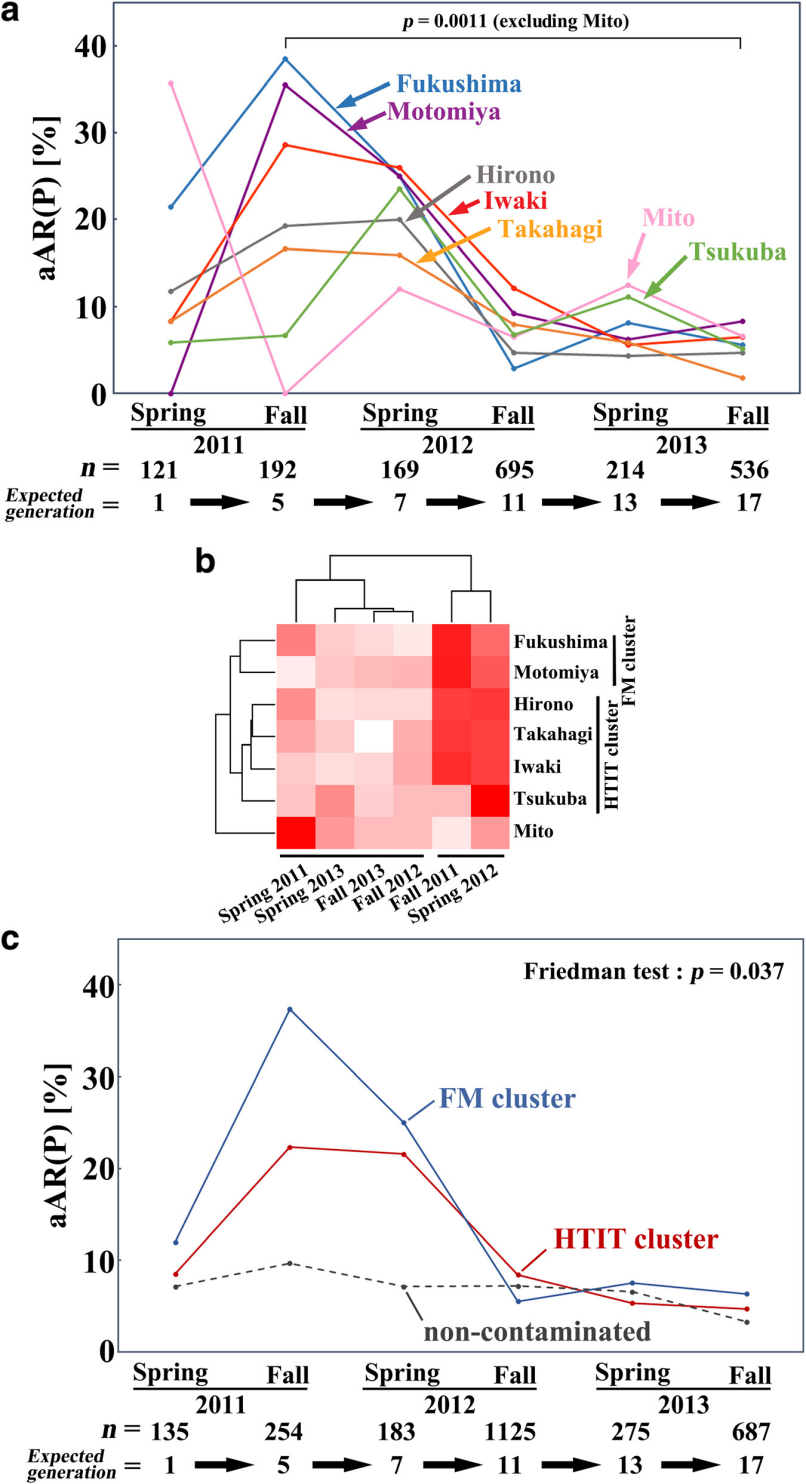
**Dynamics of the adult abnormality rate (aAR) of the P generation from 7 localities in 2011–2013. (a)** The aAR changes over time in 3 years. Statistically significant differences are indicated between the fall of 2011 and the fall of 2013 (*p* = 0.0011, Scheffe test) after the exclusion of Mito. The total number of individuals collected in the 7 localities (*n*) and the expected number of generations after the nuclear accident are shown. **(b)** Cluster analysis with a heat map. The cluster of Fukushima and Motomiya (FM cluster) and that of other localities (HTIT cluster) are indicated. Additionally, the cluster of the fall of 2011 and the spring of 2012 and that of other time points are indicated. Mito is positioned as an outlier. **(c)** The aAR changes of the FM and HTIT clusters over time. The aARs from other localities that can be considered non-contaminated are shown for comparison. Statistically significant differences among time points in multiple comparisons are indicated (*p* = 0.037, Friedman test). The total numbers of individuals collected in all localities, including the ones from non-contaminated localities (*n*), are indicated.

We monitored the aAR of field-collected adults from the 7 localities in 6 sampling attempts over 3 years. We first plotted the raw aAR data from the 7 localities on a single graph (Figure 3a). Considering that the normal level of aAR is below 10% (see below and also [12]), the aAR in the spring of 2011 was higher than the normal level in Hirono, Fukushima, and Mito. An increase in the fall of 2011 was then observed in many localities except Tsukuba and Mito. Notably, the localities with high levels of pollution, i.e., Fukushima and Motomiya, showed a clear peak in the fall of 2011, approaching 40%. The localities with intermediate pollution levels, i.e., Iwaki, Hirono, and Takahagi, showed similarly high aARs both in the fall of 2011 and in the spring of 2012. In contrast, the locality with the lowest contamination level, Tsukuba, peaked later, in the spring of 2012. All localities showed normal aAR levels in the fall of 2012 and afterwards. However, we noticed that Mito behaved differently from the rest, at least during the spring and fall of 2011, which was apparent in the cluster analysis (Figure 3b) and the multidimensional scaling plot (Additional file 2: Figure S1). Based on these results, we excluded Mito as an outlier from the subsequent analyses.

After excluding Mito, we found statistically significant differences between the peak time-point (i.e., the fall of 2011) and the latest time-point (i.e., the fall of 2013) (*p* = 0.0011; Scheffe test), supporting the visual interpretations of the dynamics discussed above (Figure 3a). The cluster analysis revealed that the 6 localities were segregated into two clusters: the Fukushima-Motomiya cluster (FM cluster) and the cluster including Hirono, Takahagi, Iwaki, and Tsukuba (HTIT cluster) (Figure 3b). The FM cluster corresponded to the inland cities relatively far from the ocean (but heavily contaminated) to the northwest of the FNPP, and the HTIT cluster corresponded to the coastal cities (but not affected by Tsunami) along the Pacific Ocean to the south of the FNPP. The cluster analysis also revealed two time-point clusters: the cluster containing the fall of 2011 and the spring of 2012 and the cluster containing the rest of the time points (Figure 3b), suggesting that major biological changes occurred during the fall of 2011 and the spring of 2012 in Fukushima and Motomiya.

Based on the clustering results, we then calculated the aAR values for the FM and HTIT clusters of all the combined samples from the included localities, which were plotted against time (Figure 3c). As expected, the FM cluster showed a higher aAR than the HTIT cluster except in the fall of 2012. The FM cluster showed a steep decrease in the spring of 2012 and also in the fall of 2012, but the HTIT cluster showed a large decrease only in the fall of 2012, after which the aARs were at normal levels in both clusters. The samples from other localities (excluding the major 7 localities) that were minimally contaminated (Additional file 1: Tables S1-S6) were plotted together with the FM and HTIT clusters (Figure 3c). Using these 3 groups, statistically significant differences were found among different time points in multiple comparisons (*p* = 0.037; Freidman test). Because these other localities were at the minimal contamination level, we found that the aARs of the normal populations were largely below 10%, ranging from 0% to 13.5% with a median of 6.4%. Similar results have been shown in a previous study [12], where the aARs of the field-collected samples had a mean value of 8.2%.

**Figure 4:**
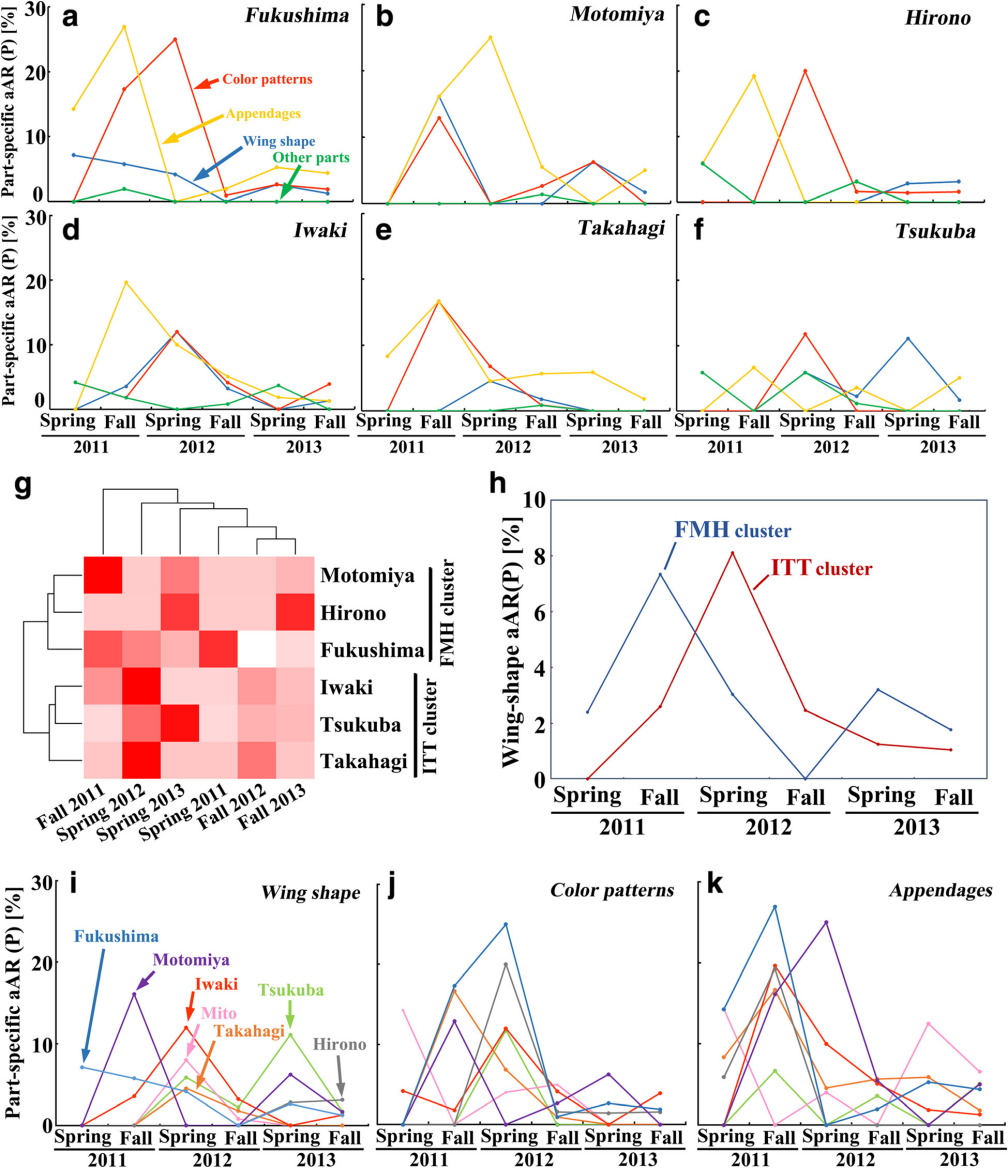
**Dynamics of the part-specific adult abnormal rate (aAR) of the P generation from 6 localities in 2011–2013. (a**-**f)** Part-specific aARs for wing shape, wing color patterns, appendages, and other parts in 6 localities. **(g)** Cluster analysis with a heat map for the wing-shape aARs. Two clusters (the FMH cluster and the ITT cluster) are indicated. **(h)** Dynamics of the wing-shape aARs in two clusters. The FMH cluster and the ITT cluster are shown. **(i**-**k)** Part-specific aARs for wing shape, wing color patterns, appendages, and other parts in 6 localities.

The adult abnormalities were classified into four different categories (wing shape, wing color patterns, appendages, and other parts), and their changes over time were examined in the 6 different localities (Figure 4a-f). Overall, it seemed that abnormalities in the appendages occurred earlier, followed by abnormalities in wing color patterns and wing shape, especially in the relatively highly contaminated localities. The relatively small level of wing-shape abnormalities may be due to the limited mobility of such individuals, resulting in a lower probability of being captured. A cluster analysis on the wing shape abnormalities revealed two clusters: one cluster included Fukushima, Motomiya, and Hirono (FMH cluster), and the other cluster included Iwaki, Takahagi, and Tsukuba (ITT cluster) (Figure 4g). The incidence of abnormality in the FMH cluster peaked in the fall of 2011, whereas abnormality in the ITT cluster peaked in the spring of 2012 (Figure 4h), showing a delay of peaking in the low-contamination localities.

We then plotted the four categories of the abnormalities individually throughout the 3-year period. The incidence of wing shape abnormality was irregular and sporadic over time (Figure 4i). Abnormalities in wing color patterns peaked mainly in the spring of 2012 (Figure 4j), and abnormalities in the appendages peaked mainly in the fall of 2011 (Figure 4k). The abnormalities of the other parts were quite irregular and sporadic (not shown). Wing shape abnormalities may be behaviorally lethal or sublethal, and such abnormal individuals cannot be captured frequently by us in the field, resulting in the sporadic and low-level occurrence. In contrast, abnormalities in appendages and color patterns may be less lethal, and individuals with such abnormalities can still be captured by us in the field, resulting in the peaks at specific time-points. This interpretation is in good agreement with the results of the F_1_ generation discussed later.

**Figure 5:**
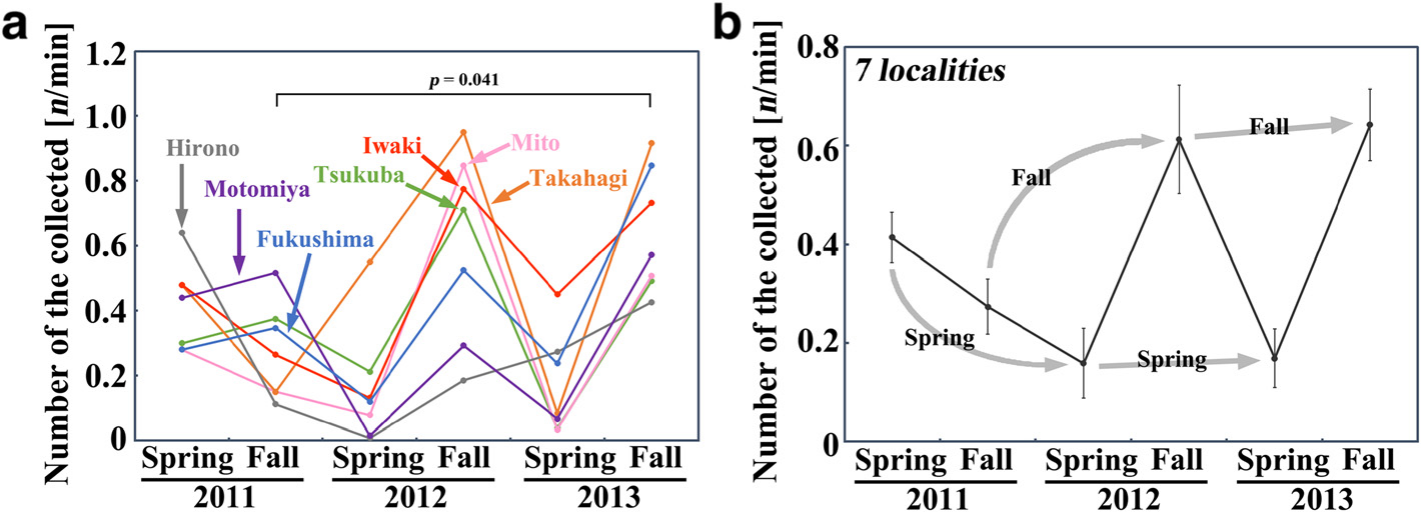
**Dynamics of the number of collected individuals per minute in 2011–2013. (a)** Seven localities over 3 years. **(b)** Seven localities combined over 3 years. Arrows indicate changes over 3 fall points or 3 spring points that are comparable to one another. Error bars indicate the mean ± SE.

Consistent with these results, the number of individuals caught per minute in the field was low in both the fall of 2011 and in the spring of 2012 (Figure 5a, b). These dynamics should be interpreted with caution because it is likely that the adult density is generally low in the spring and gradually increases toward the summer every year, at least in Tokyo [16] and most likely in the entire Tohoku and Kanto districts. Moreover, the number of individuals caught per minute is also affected by environmental factors including the weather of the day. Nevertheless, the relatively low number of individuals in the fall of 2011 was notable compared with the number in the fall of 2013 (*p* = 0.041; Scheffe test). For example, in Hirono, we were able to collect only very small number of individuals in the fall of 2011 and in the spring of 2012 despite the fine weather.

In summary, the radiation levels peaked first in the spring of 2011, followed by an increase in the aARs and a decrease in the number of individuals in the fall of 2011. Then, the aAR decreased and the number of individuals increased back to normal levels. This chronological order supports the normalization hypothesis, rejecting the extended mortality hypothesis and the extinction hypothesis (see Introduction).

### The tAR and aAR dynamics of the reared offspring

**Figure 6:**
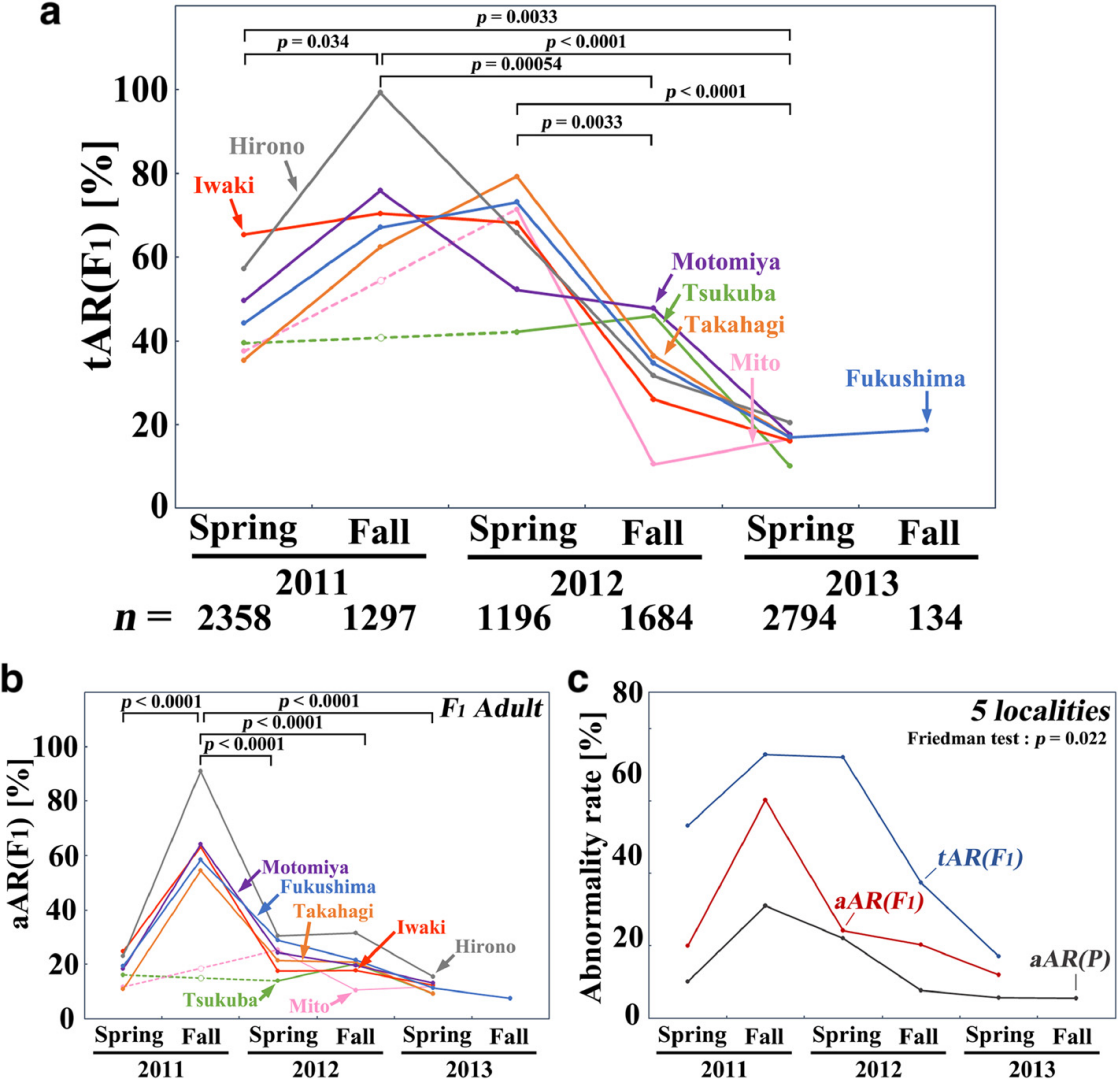
**Dynamics of the abnormality rate (AR) in the offspring (F**_**1**_ **) generation in 2011–2013. (a)** The tAR(F_1_) changes of 7 localities. Statistically significant differences between two time-points in multiple pairs (Scheffe test) or multiple comparisons (Friedman test) are indicated in all panels in this figure. Time points with a lack of data are indicated by blank circles with dotted lines (also in other panels in this figure). **(b)** The aAR(F_1_) changes in 7 localities. **(c)** The tAR(F_1_), aAR(F_1_), and aAR(P) changes in 5 localities combined.

We next monitored the total AR (tAR) of the offspring (F_1_) generation from the adults caught in the 7 localities (Figure 6a; Additional file 3: Table S7). The tAR includes the deaths in the larval, prepupal, and pupal stages and the abnormalities in the surviving adults, which could be examined by rearing the offspring in the laboratory. The tARs peaked mostly either in the fall of 2011 or in the spring of 2012, similar to the results of the aAR of the parent generation shown above, with a significant difference between the fall of 2011 and the spring of 2013 (*p* < 0.0001; Scheffe test with Benjamini-Hochberg correction [17]) and between other two time-points. However, we did not obtain clear clusters in the cluster analysis and the multi-dimensional scaling (not shown), suggesting that the tAR patterns over time were similar among the localities monitored. The decrease of the tAR was relatively slow: the tAR levels decreased back to normal in the spring of 2013, taking more time than the aAR of the parent generation. Another noteworthy point was that the levels of the tARs were rather high. These results suggest a transgenerational character of abnormalities, at least from the P to F_1_ generations, throughout this time period and also suggest that selection against abnormal individuals occurred in the field throughout this time period.

We also plotted the aAR of the F_1_ generation (Figure 6b). The aARs of the F_1_ generation had higher values and peaked more sharply in the fall of 2011 than the aAR of the parent generation in all seven localities. For comparison, we integrated all 5 localities (excluding Mito and Tsukuba due to a lack of monitoring data in the fall of 2011) by considering that all samples were collected from a single Tohoku-Kanto area, and we plotted the aAR of the P and F_1_ generations and the tAR of the F_1_ generation together (Figure 6c). All 3 ARs showed an overall similar pattern: a peak in the fall of 2011 and a subsequent decrease. However, it is notable that the tAR peak was much higher than the peaks of aARs and that the tAR peak in the fall of 2011 extended to the spring of 2012. The aARs of the P and F_1_ generations were similar to each other in that they both showed a single peak in the fall of 2011, but they were clearly different in magnitude; the aAR of the F_1_ generation was much higher. These results confirmed the idea that the wild populations of this butterfly produced many abnormal individuals that were selected out before appearing as viable adults in the field.

As in the previous analysis of the parent generation, the adult abnormalities were classified into 4 categories (wing shape, wing color patterns, appendages, and other parts), and the changes in the aARs over time were examined (Additional file 4: Figure S2). The levels of aARs were especially high for the wing shape in the fall of 2011 (Additional file 4: Figure S2a) compared with the wing color patterns (Additional file 4: Figure S2b), the appendages (Additional file 4: Figure S2c), and the other parts (not shown).

**Figure 7:**
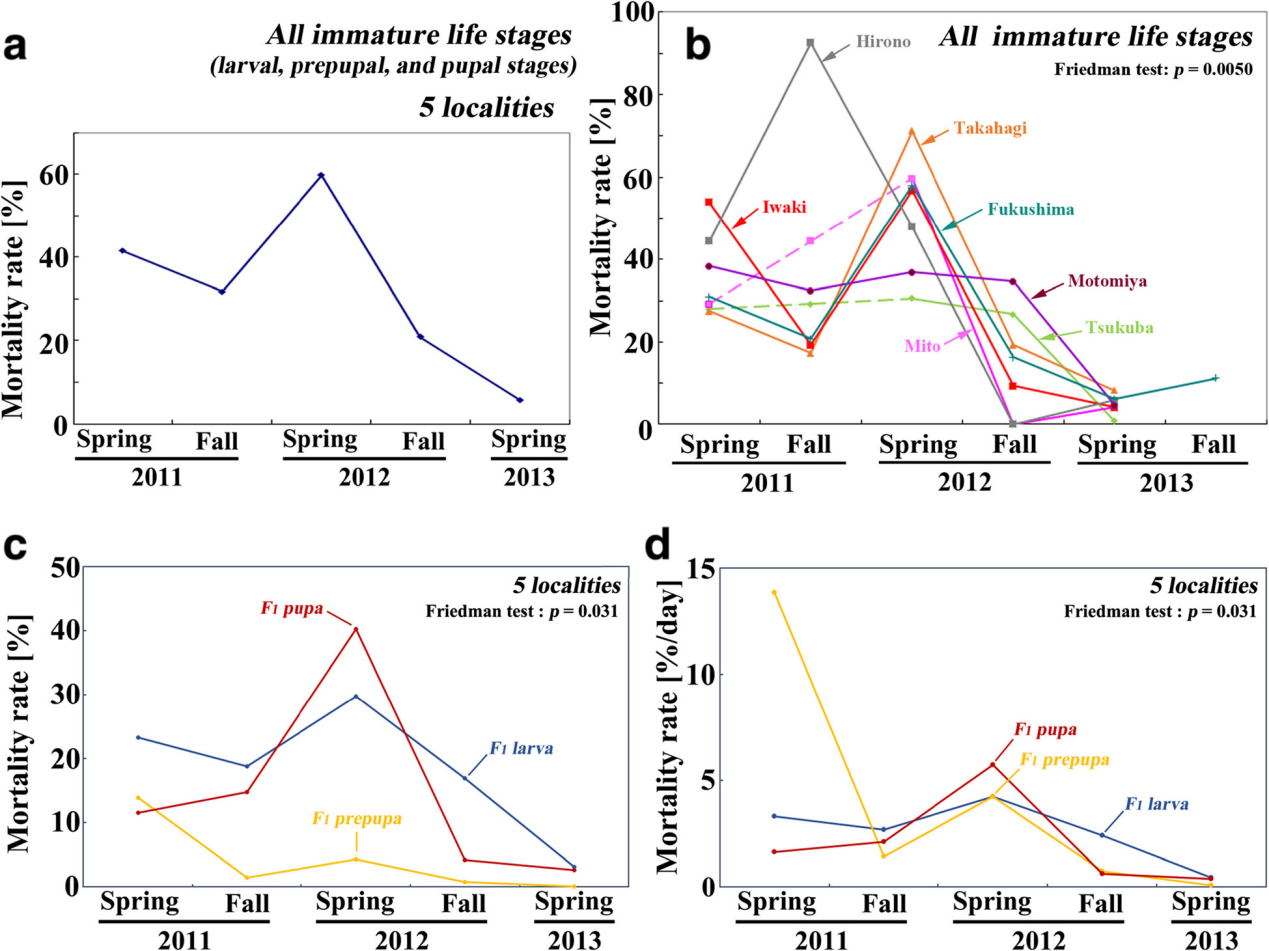
**Dynamics of the mortality rate (MR) in the offspring (F**_**1**_ **) generation in 2011–2013. (a)** Dynamics of MR throughout immature life stages (i.e., larval, prepupal, and pupal stages) of 5 localities combined. **(b)** Locality-based dynamics of MRs throughout immature life stages. **(c)** Dynamics of larval, prepupal, and pupal MRs of 5 localities combined. **(d)** Dynamics of larval, prepupal, and pupal MRs per day of 5 localities combined.

The dynamics of the mortality rate (MR) added further insights into what occurred in the polluted area. When all 5 localities were combined as samples from a single collection area, the MR throughout immature life stages (i.e., larval, prepupal, and pupal stages) showed the highest in the spring of 2012, scoring approximately 60%, and decreased afterwards to the normal level in the spring of 2013 (Figure 7a). When 5 localities were examined separately, Hirono notably scored more than 90% in the fall of 2011 (Figure 7b), suggesting that the large-scale death occurred in Hirono at that time. Takahagi, Mito, Fukushima, and Iwaki scored more than 50% in the spring of 2012.

When the MRs of developing life stages (the larval, prepupal, and pupal MRs) were examined separately in all 5 localities combined, the pupal MR was highest in the spring of 2012 and decreased sharply afterwards (Figure 7c). When the stage-specific MRs were simply divided by the number of days for each stage, prepupal death was notable in the spring of 2011 (Figure 7d). The dying stage may be different between 2011 and 2012. The main selection stage was likely the prepupal stage in the spring of 2011 and then moved to the pupal and larval stages afterwards. Because we reared these larvae, prepupae, and pupae under standard laboratory conditions, where no artificial radionuclides were detected, and in the absence of any predators, the selection pressures against these immature stages were likely to be more severe in the field than in the laboratory.

We then examined the dynamics of the stage-specific MRs in 7 different localities (Additional file 5: Figure S3). Most locations showed peak larval mortality in the spring of 2012, with the exception of Hirono, at which larval mortality peaked in the fall of 2011, and of Motomiya, at which larval mortality peaked in the fall of 2012 (Additional file 5: Figure S3a). At the prepupal stage, the highest level of mortality was found in the spring of 2011 in most localities, again with the exception of Hirono, at which prepupal mortality peaked in the fall of 2011 (Additional file 5: Figure S3b). At the pupal stage, most localities exhibited peak mortality in the spring of 2012, again with the exception of Hirono, at which mortality peaked in the fall of 2011 (Additional file 5: Figure S3c).

### Correlations of ARs with the ground radiation level and with the distance

To investigate the possible causal involvement of radioactive pollution released by the Fukushima nuclear accident in the AR dynamics in 2011–2013, we examined correlations of ARs with the ground radiation dose at the collection sites and with the distance from the FNPP, although a similar analysis has already been performed for the 2011 data in previous papers [9,12,14]. Radioactive materials released early, which mainly contained short-lived radionuclides, were dispersed in a concentric fashion, whereas radioactive materials released later, which mainly contained cesium, were dispersed mainly toward the northwestern cities [2]. Therefore, our categorization may help to distinguish the effects.

**Figure 8:**
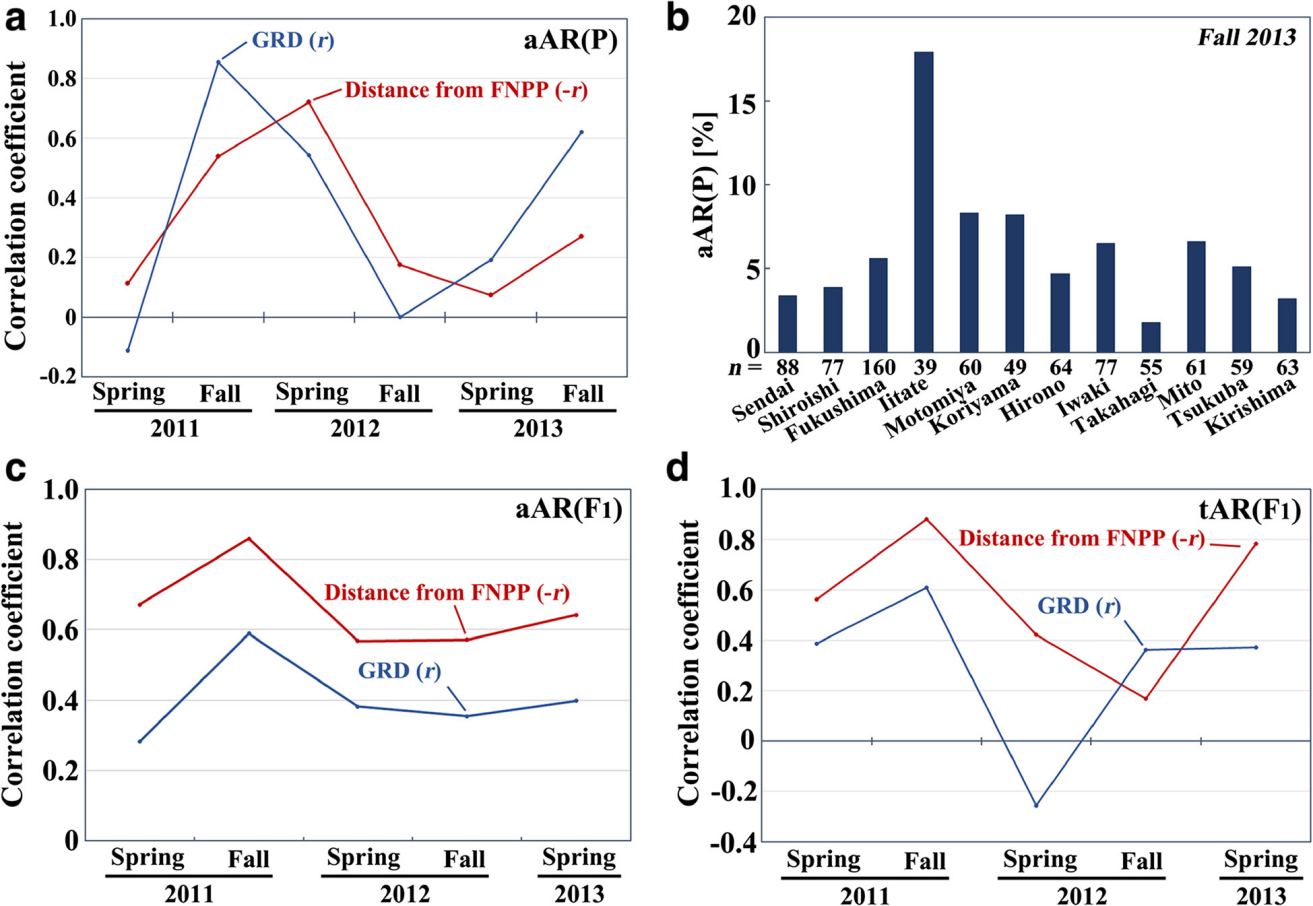
**Correlation analysis of aAR(P), aAR(F**_**1**_ **), and tAR(F**_**1**_ **). (a)** Dynamics of Pearson correlation coefficients (*r* or –*r*) between the aAR(P) of the field-collected samples (the P generation) and the ground radiation dose (GRD) or the distance from the FNPP in 2011–2013. **(b)** The aAR(P) levels of 12 localities in the fall of 2013. **(c)** Dynamics of Pearson correlation coefficients (*r* or –*r*) between the aAR(F_1_) of the laboratory-reared samples (the F_1_ generation) and the ground radiation dose (GRD) or the distance from the FNPP in 2011–2013. **(d)** Dynamics of Pearson correlation coefficients (*r* or –*r*) between the tAR(F_1_) of the laboratory-reared samples (the F_1_ generation) and the ground radiation dose (GRD) or the distance from the FNPP in 2011–2013.

In the P generation, changes in Pearson correlation coefficients over the 6 time points between the aAR level and the ground radiation level and between the aAR level and the distance were similar to each other, but with a slight delay in the distance (Figure 8a). Correlation was strongest in the fall of 2011 for the ground radiation and in the spring of 2012 for the distance, both of which then decreased toward the fall of 2012 or toward the spring of 2013.

Unexpectedly, the coefficients then increased again in the spring of 2013 for the ground radiation and in the fall of 2013 for the distance. To understand what occurred, scatter plots were examined (Additional file 6: Figure S4). The relatively high correlation of aAR with the ground radiation dose in the fall of 2011 was disrupted mainly because of the fast decrease of the aARs in the high-radiation localities (Additional file 6: Figure S4a). By the fall of 2013, the aARs in the low-radiation localities decreased, recreating relatively high correlation again, but with much lower aARs. Similar dynamics, although less clear, were observed in the distance correlation (Additional file 6: Figure S4b).

To understand the latest state of the aARs, we examined the aARs of the adult samples that we collected in the fall of 2013 from 12 localities (Figure 8b). The aARs were relatively low in most localities but relatively high in Motomiya and Koriyama and very high in Iitate, confirming the origin of the relatively high correlation in the fall of 2013.

Similar dynamics were obtained in the F_1_ generation with the aARs (Figure 8c) and with the tARs (Figure 8d), confirming the trends obtained in the P generation. Scatter plots also showed a similar trend (Additional files 7, 8: Figure S5, S6). However, the peak time difference, observed in the P generation, between the ground radiation dose and the distance (Figure 8a) was not observed in the F_1_ generation (Figure 8c, d).

## Discussion

In the present study, we described the spatial and temporal dynamics of the ARs of the pale grass blue butterfly in the first 3 years after the Fukushima nuclear accident. The field sampling attempts were conducted in 7 localities (although one was later considered an outlier) 2 times a year. There are no long-term study on the biological effects in the Fukushima accident from the beginning of the accident covering several localities except this paper, and nothing similar will be published in the future. Importantly, in addition to the aARs of the field populations, we recorded the tARs and aARs of the offspring generation, which were obtained under standard rearing conditions with neither external nor internal radiation exposure from artificial radionuclides. Therefore, we believe that the present study is invaluable to understand biological effects of the accident.

We witnessed the time course in which the disturbed populations regained their normal AR levels. The ARs do not consider the degrees of abnormality in a given individuals, but we believe that the degrees of abnormality parallel the ARs over 3 years. Therefore, we assert that the normalization hypothesis, but not the extended mortality hypothesis and the extinction hypothesis, is correct (see Introduction).

Because the spatial and temporal dynamics of the ARs of the parent (P) and offspring (F_1_) generations were largely similar to each other, we assert that these results are reasonably precise and accurate descriptions of the genetic and physiological effects that were accumulated over generations. In this study, we do not know if the observed transgenerational effects are genetic or physiological. Because maternal effects do not have to be genetic, we do not know if DNA of the P generation was damaged. In any case, it should be noted that if there was no transgenerational effect representing the heritable damage from the parent generation, the tAR of the offspring generation would be always at the normal level because the offspring generation was obtained and reared under non-contaminated laboratory conditions. The experimental reproduction (tAR and aAR of the F_1_ generation) of the field data (aAR of the P generation) are highly important, especially in those studies that examine a rare field event. It should also be noted that because we obtained tARs by rearing larvae in Okinawa using non-contaminated leaves, the actual tARs of the field populations in polluted areas would be considerably higher than those of the laboratory-reared samples, considering the continuous and simultaneous external and internal radiation exposures in the field. Indeed, our previous studies demonstrated independent but similar effects of external and internal exposures in terms of deaths, morphological abnormalities, and small wing size in this butterfly [9,12,14].

We provided evidence that the butterfly populations were severely disturbed, especially in the fall of 2011 and the spring of 2012. The AR levels in the fall of 2011 and the spring of 2012 were notably higher than those at other time points, both in the parent and offspring generations. Cluster analysis revealed Fukushima and Motomiya to be clustered together based on the aAR(P) and Fukushima, Motomiya, and Hirono based on the wing shape abnormalities. These results imply that radioactive contamination in these 2 or 3 localities had deleterious impacts on butterflies. The status of Hirono is somewhat ambiguous but understandable in that Hirono is located more closely to the FNPP than other localities examined here and thus was more contaminated by high-energy radionuclides [2]. These results also imply that wing shape is likely sensitive to radiation exposure.

It is interesting that the temporal dynamics of the high- and low-pollution localities differed in the magnitude of peak AR but shared both the timing of the AR decline and the level of the steady state (i.e., normal state) in 2013. That is, the population accumulated the adverse effects transgenerationally in the first year but regained normality by the third year in all localities. Earlier achievements of the steady state in the low-pollution localities were not clearly observed. The reasons for this are not known, but these data compellingly indicate that the butterfly populations in the localities that we surveyed were highly disturbed in 2011 and regained normality by 2013.

In examining the mortality rates of the F_1_ generation during the larval, prepupal, and pupal stages, we noticed that the prepupal death rate in the spring of 2011 was notably high. This may be considered an acute effect of radiation exposure. We also noted peaks of the mortality rates of all 3 stages in the spring of 2012 but not in the fall of 2011, as might have been expected from the dynamics of aAR(P) and aAR(F_1_). This may be a transgenerational accumulation of damage or may be considered a chronic effect of radiation exposure. These acute and chronic effects may be independent. Morphological abnormalities in adult individuals, frequently seen in the fall of 2011, can be interpreted not to be extremely severe, in that these individuals survived to the adult stage and that the most severe symptom was death, frequently seen in the spring of 2012.

What is the cause for this transgenerational disturbance widely seen in the Tohoku and Kanto districts that occurred specifically in 2011? The present field-based study is necessary, but not sufficient, to prove the causal role of the Fukushima Dai-ichi NPP accident in this disturbance of the pale grass blue butterfly. Nevertheless, we observed high correlations between the ARs and the ground radiation dose (i.e., dose dependence) and between the ARs and the distance from the FNPP. These results do not prove but clearly suggest the causality of the radioactive pollution to the temporal and spatial AR dynamics, which is even more compelling when we consider the preceding studies on this butterfly [9-14].

Higher correlations of ARs with the distance than with the ground radiation dose that were obtained in the F_1_ generation can be understood if the biological impacts by the early burst mainly containing short-lived species were severer than those by the later release mainly containing cesium. The exceptional behavior of Hirono may also be due to the early exposure to high-energy radionuclides because of its proximity to the FNPP (approximately 20 km) [2]. Thus, the time course of deaths proceeded from prepupae (and larvae to a lesser extent) in the spring of 2011 to pupae in the spring of 2012. The prepupal stage is short, spanning approximately just one day, but it is a very important stage for metamorphosis. The impacts of radiation are especially severe at the prepupal stage probably because of highly frequent cell division during metamorphosis with high metabolic activity.

In the case of the Chernobyl accident, a long-term study has been conducted using a small mammal, the bank vole [18]. Chromosomal aberrations and embryonic lethality did not cease even after 22 generations in 10 years [18]. We believe that differences from the butterfly dynamics may be because of differences in generation time, ecological status, amount and species of radionuclides released, and other factors.

Assuming that the population disturbance observed above was caused by the radioactive materials from the collapsed FNPP, we propose two possible reasons for the falling phase of the ARs: first, due to a decrease in the pollution level itself over time and second, due to adaptive evolution for resistance against radioactive stress.

The first possibility that a decrease in radioactivity levels over 3 years contributed to the recovery of the normal state of the populations appeared to be reasonable. We believe that the impacts of the early, acute exposure to the fast-decaying radionuclides that existed immediately after the burst (e.g., ^131^I and ^129^Te) were most likely higher than the chronic exposure to the slow decaying radionuclides that remained for years [14]. Furthermore, because the pale grass blue butterfly lives on or near the ground surface, especially at the larval stage, this butterfly is likely to be severely affected not only by γ-rays but also by β-rays [19]. The importance of β-rays was implicated by a high count rate detected by a GM counter in the spring of 2011 in the present study. The GM counter’s count rates quickly decreased afterwards. A similar but milder decrease was observed in the radioactivity level detected by a scintillation counter. However, it is important to note that the radiation level at the high-pollution localities in the fall of 2013 was still much higher than the levels at the low-pollution localities in the spring of 2011. Nevertheless, the ARs of all localities showed similar temporal dynamics over 3 years and converged to a similar, normal level in 2013. Therefore, the decrease of the ARs to the normal level cannot solely be attributed to the decrease in radiation levels.

The second possibility states that a decrease of ARs was a result of adaptive evolution through natural selection against abnormal adults and radiation-susceptible larvae, prepupae, and pupae in the field. Because the tAR and aAR of the offspring generation reared under standard conditions with no artificial external and internal radiation exposure were much higher than the aAR of the parent generation, the pollution likely acted as a source of strong selective pressure for this butterfly. Only normal and nearly normal individuals could be collected in the field, and this tendency appeared to be higher in more polluted areas. Consistent with this idea, the number of adult individuals caught per minute, which represents the population density, appeared to have decreased dramatically in the fall of 2011 and the spring of 2012. The AR peaks were observed not in the spring of 2011 but in the fall of 2011 and the spring of 2012. This delay from the time of the accident would be best explained by the biological accumulation of adverse effects, followed by a falling phase that may be explained by adaptive evolution through the process of natural selection. Adaptation to radiation stress has been reported in the literature. For example, transgenerational developmental responses to radiation have been documented in grasshoppers in Chernobyl [20], and possible adaptation to oxidative stress in birds as a consequence of the Chernobyl accident has been reported recently [21]. In regions with high natural radiation levels, radiation resistance has been observed in *Drosophila* [22] and other organisms [23]. What is novel in the present study is the real-time documentation of the spatial and temporal dynamics of ongoing evolution.

The migration of animals from low-pollution areas to high-pollution areas may be another issue to consider. Although the mobility of this butterfly species is rather limited [12,14], it could migrate many kilometers over generations, and the increase of the population (i.e., the number collected) after a decline may partly be explained by immigrants from less affected areas. However, the contribution of immigrants to the decrease of the ARs would likely be small because immigrants from non-polluted areas would also face a high-radiation environment, resulting in high tARs of the immigrants, similar to the native residents.

Demonstrating the biological effects of long-term low-dose radiation exposure (or chronic exposure) due to nuclear pollution has scientific and political complications [24]. In the case of the Chernobyl accident, a paucity of scientific studies covering the early years after the accident generated confusion about the biological impacts of the accident [25]. The Fukushima case was the first opportunity in the history of mankind to rigorously study the biological effects of a large-scale nuclear accident from the very beginning of the accident. It should be recognized that the results of this type of study are heavily dependent on the time period of a given study and on generation time, radiation resistance or susceptibility, and evolvability of the biological species of interest. For example, this study retrospectively demonstrated the importance of an accurate description immediately after the nuclear accident to understand the biological impacts of the accident. In other words, the present study implies that any study that did not cover the early years after a leakage of radioactive materials may have overlooked the real biological impacts because of a relatively quick adaptation process, especially in organisms that have a short-generation time, such as multivoltine insects. Likewise, any study on organisms that have a long generation time, including large mammals, may have also overlooked the biological impacts because the transgenerational effects are difficult to detect.

The pale grass blue butterfly has a short generation time, and the AR peak in the fall of 2011 corresponds to the fifth generation after the accident. The rise and fall of the ARs was completed in approximately 2 years, in 11–13 generations. By extrapolation, an organism that has a generation time of one year may show the worst effects in 2016. Assuming that the human generation time is 20 years, the adverse effects in this most intelligent species would increase gradually toward 2111, if our results in the butterfly can be applied directly to humans.

If the biological phenomena we report here involve adaptive evolution in the polluted environment, it means that we witnessed the real-time adaptive evolution for radiation resistance in the pale grass blue butterfly. This evolutionary process is likely driven by natural selection, as suggested by the difference in aARs between the parent and offspring generations. The radioactive pollutants themselves are harmful to organisms, which necessitates natural selection, although it is ironic to call this anthropogenically mediated selection “natural”.

Precisely recorded real-time evolution in the field is still rare, but the well-known textbook version of evolution is the case of the peppered moth, *Biston betularia*, which showed industrial melanism [26-30]. Furthermore, a case of interesting real-time evolution has recently been published [31]. It is interesting to note that we previously demonstrated that the pale grass blue butterfly showed evolutionary changes in its color patterns at its northern range margin [32-34]. This case occurred in geographically different regions and is clearly distinguishable, phenotypically and mechanically, from the Fukushima case. In the former, specific color patterns evolved [34], whereas in the latter, radiation resistance evolved, although the resistance that evolved in the population in the polluted localities should be demonstrated physiologically by laboratory experiments in subsequent studies.

## Conclusions

In this study, we presented the spatial and temporal dynamics of ARs in the first 3 years of the Fukushima nuclear accident, which clearly suggest the nuclear accident’s causation of the biological changes. The rise and fall of the ARs were recorded, which is likely due not only to the decrease of the radioactive materials in the environment but also to adaptive evolution of the butterfly through natural selection. Our study clearly showed the importance of prompt and continuous field monitoring after a nuclear accident.

## Methods

### Ethics

No specific permissions were required to sample the pale grass blue butterfly and its host plant in Japan.

### Field work

We collected adult butterflies twice a year (spring and fall) in three years (2011–2013) mainly from the northern Kanto district and the southern Tohoku district, Japan (Figure 1). The exact locations and days of collection are listed in Additional file 1: Tables S1-S6. Typically, we visited the 7 localities (Tsukuba, Mito, Takahagi-Kitaibaraki, Iwaki, Hirono, Motomiya, and Fukushima) in 5 days, mostly from the southern to northern cities. Because Takahagi and Kitaibaraki are adjacent cities, close to each other, we considered them a single locality called “Takahagi”. These localities were not affected by Tsunami at all. Changes in human activities after the earthquake and the nuclear accident in these localities are unlikely to change habitats for the pale grass blue butterfly except high levels of radioactive pollution, because people did not abandon their land and livelihood in these cities.

Four people (three in the spring of 2013) carried out butterfly collecting in a 20-minute period (as a search time unit) at a collection site using a small net (30 cm in diameter). The butterflies caught in the net were transferred to screw-capped columnar tubes (13 mm in inner diameter and 50 mm in height) and were then stored in a cooler box (kept at less than 10°C) until they reached the laboratory in Okinawa and released into a cage. Artificial nectar (diluted POCARI SWEAT; Otsuka Pharmaceuticals, Tokyo, Japan) was given to these butterflies in tubes and in cages when necessary. For comparison, we also collected butterflies in the Okinawa, Kagoshima, Fukuoka, Yamaguchi, Ehime, Hyogo, and Aomori prefectures (Figure 1; see Additional file 1: Tables S1-S6).

### Dose measurement

At the time of butterfly collection, the ground radiation dose at the collection sites was measured using an Aloka TGS-146 or TGS-133 Geiger-Müller (GM) survey meter (Hitachi Aloka Medical, Tokyo, Japan) for count rates of β-rays (and, less efficiently, γ-rays) and using an Aloka TCS-161 or TCS-172 scintillation survey meter (Hitachi Aloka Medical, Tokyo, Japan) for dose rates of γ-rays. These survey meters were calibrated in April 2011, 2012, and 2013 before field surveys and also in November 2011 (or in November 2008 and December 2011 for TCS-161 with operation check every month) using ^226^Ra as a standard radiation source. The DoseRAE 2 (RAE Systems, San Jose, CA, USA) was also used for dose rates of γ-rays. Its outputs were compared with those of the Aloka TGS-146 in November 2011. Their outputs were found to be almost identical when they were placed far (beyond approximately 50 mm) from a standard ^226^Ra radiation source. We identified a host plant that was nearest to a butterfly collection point, on which radioactivity was measured. Measurements were made at 0 cm (on the ground surface), 30 cm, and 100 cm above the ground for 30 sec. From that point, two points 1 m away on both the right and left sides were also measured, and these values were averaged for statistical analysis.

### Morphological examination

In the laboratory, field-collected butterflies were sexed and checked for morphological abnormalities under a standard stereo microscope. We examined morphological abnormalities of the following structures: wing shape, including venation; color patterns of the ventral wings; appendages, including forelegs, midlegs, hindlegs, antennae, proboscis, and palpi; compound eyes; and other gross structures in the head, thorax, and abdomen. The numbers of abnormal individuals were obtained for four different groups: wing shape, wing color patterns, appendages, and other parts. An abnormality rate for each group was calculated by dividing the number of abnormal individuals by the number of individuals that were safely subjected to morphological examinations. Thus, individuals that had physically worn structures that made it difficult to examine the original morphological structures were excluded from the denominator. We also excluded the possible color pattern aberrations that were similar to the temperature-shock-induced color patterns (TS types), despite the fact that the aberrant color patterns are unlikely to be induced by temperature shock, at least in the fall samples. We identified the individuals with color patterns that considerably differ from the basic patterns of this butterfly [35] as color pattern abnormalities, namely, disarrangement of spots (DS), reduction of spots (RS), loss of spots (LS), fusion of spots (FS), ectopic spot (ES), dislocation of spots, and enlargement of spots (not many spots, being different from the temperature-shock type), some of which (indicated by two-letter codes) were shown in a previous mutagenesis experiment [35]. The abnormality rate we obtained this way may be an underestimate because we scored a given structure “abnormal” only when it differed considerably beyond individual variations of normal morphology.

### Egg collection and butterfly rearing

We followed our standard rearing methods [15] with a few minor modifications [6-8,35]. A pool of apparently healthy females caught in the field of the seven localities was used to collect eggs. We successfully obtained the F_1_ generation from the major localities. An exception was the fall of 2013, when we obtained the F_1_ generation only from the females collected from Fukushima, simply due to our technical failure. Most females from the field were not virgin, but just in case, we put some males in the cages with females and encouraged mating to ensure fertilization. The number of individuals used for the egg collection process is shown in Additional file 2: Table S7. When females died in this period, another female was added to the cage when available. Larvae were fed non-contaminated leaves of the host plant *Oxalis corniculata* from Okinawa to exclude the influence of internal radiation exposure.

The first-instar larvae were reared in a transparent plastic cup container (100 mm in diameter, 55 mm in depth) 3 or more days after they hatched. The third- or fourth-instar larvae were reared in a transparent plastic square container (140 mm in width, 140 mm in depth, 55 mm in height). The container was cleaned and the larvae were fed fresh leaves every day or every other day, depending on the cleanliness and larval appetite. We counted the number of individuals at the second or third instar, after which we started recording the larval deaths and other abnormalities as they grew. We obtained a mortality rate at the larval, prepupal, and pupal stages and an abnormality rate at the adult stage, as in the field-caught adults. The mortality rate per unit time was calculated by dividing the original mortality rates by the relative mean larval period of 7 d (after the number of individuals were counted), the relative mean pupal period of 7 d, and the relative mean prepural period of 1 d, which were based on the data of normally developed individuals from the Tsukuba F_1_ generation in the fall of 2011. We also recorded the date of prepupation, pupation and eclosion and obtained the peak pupation date, the half-pupation date, the peak eclosion date, and the half-eclosion date (not shown). Successfully eclosed adults were defined as adults with all four wings completely detached from the pupal case.

### Statistical analysis

We used the statistical software package R, version 3.0.2 (The R Foundation for Statistical Computing, Vienna, Austria). The Pearson correlation coefficient *r* and its associated *p*-value were obtained to examine relationships between two variables. For the cluster analysis, the ward linkage method was used with Canberra distance. A heat map was created together with the clustering results. The ward linkage method with the Canberra distance was also used for the classical multidimensional scaling (principal coordinate analysis). Based on these results, Mito was excluded as an outlier. The reasons for its unusual behavior are not clear, but we obtained information that insecticides were widely sprayed at the collection site (the Kairakuen Park) a few days before the collection dates for the routine maintenance of lawns. Localities that showed ground radiation doses less than 0.13 μSv/h (being similar to natural radiation levels of some localities in Japan) were defined as minimally contaminated or non-contaminated for the sake of discussion and were used for comparison with the contaminated localities. We performed Scheffe tests (with the Benjamini-Hochberg correction [17] when necessary) and Freidman tests for collection time points. For the examination of the number of collected individuals per minute, the trials with no individuals collected were excluded from analysis. Weather conditions affect the efficiency of collection, but we did not correct the original data, assuming that the number of individuals caught per trial will be averaged after many trials with variable weather conditions.

## Additional files

Additional file 1:
**Collection localities. Table S1.** Spring of 2011. **Table S2.** Fall of 2011. **Table S3.** Spring of 2012. **Table S4.** Fall of 2012. **Table S5.** Spring of 2013. **Table S6.** Fall of 2013. Additional file 2: Figure S1. A multidimensional scaling plot for the adult abnormality rate (aAR) of the P generation in 7 localities in 2011–2013. Mito is positioned far from other localities. Additional file 3: Table S7. Number of adults used for the egg collection process. Additional file 4: Figure S2. Dynamics of the adult abnormality rate (AR) in the offspring (F_1_) generation in 2011–2013 based on different categories. **(a)** Wing-shape-specific aAR(F_1_) dynamics. **(b)** Color-pattern-specific AR(F_1_) dynamics. **(c)** Appendage-specific aAR(F_1_) dynamics. Additional file 5: Figure S3. Stage-specific mortality rate (MR) in 7 different localities. **(a)** Larval MR dynamics. Broken lines indicate lack of data in the fall of 2011 (also in the next panels). **(b)** Prepupal MR dynamics. **(c)** Pupal MR dynamics. Additional file 6: Figure S4. Scatter plots of aAR(P). **(a)** Scatter plots of aAR(P) and the distance of the collection localities from the FNPP. Pearson correlation coefficients are shown together with *p*-values and the number of collection localities (*n*). **(b)** Scatter plots of aAR(P) and the ground radiation dose. Pearson correlation coefficients are shown together with *p*-values and the number of localities (*n*) that were subjected to radioactivity measurements. Additional file 7: Figure S5. Scatter plots of aAR(F_1_). **(a)** Scatter plots of aAR(F_1_) and the ground radiation dose. Pearson correlation coefficients are shown together with *p*-values and the number of localities (*n*) that were subjected to radioactivity measurements. **(b)** Scatter plots of aAR(F_1_) and the distance from the FNPP. Pearson correlation coefficients are shown together with *p*-values and the number of localities (*n*) that were subjected to radioactivity measurements. Additional file 8: Figure S6. Scatter plots of tAR(F_1_). **(a)** Scatter plots of tAR(F_1_) and the ground radiation dose. Pearson correlation coefficients are shown together with *p*-values and the number of localities (*n*) that were subjected to radioactivity measurements. **(b)** Scatter plots of tAR(F_1_) and the distance from the FNPP. Pearson correlation coefficients are shown together with *p*-values and the number of localities (*n*) that were subjected to radioactivity measurements.
