# Supplementary material for: Spatiotemporal abnormality dynamics of the pale grass blue butterfly: three years of monitoring (2011–2013) after the Fukushima nuclear accident
Source: BMC Evol Biol. 2015 Feb 10;15:15. doi: 10.1186/s12862-015-0297-1 (PMC4335452; doi:10.1186/s12862-015-0297-1)
Supplement: Additional file 1: — Collection localities. Table S1. Spring of 2011. Table S2. Fall of 2011. Table S3. Spring of 2012. Table S4. Fall of 2012. Table S5. Spring of 2013. Table S6. Fall of 2013. [file 12862_2015_297_MOESM1_ESM.pdf]

Table S1. Collection localities in the spring of 2011.

Collection date	Prefecture	City	Location	Distance from the NPP (km)	Ground radiation dose (μSv/h)	Number of collected		
						Total	Male	Female
13-18 May	Miyagi	Shiroishi	Aoki, Fukuokafukaya	76.7	0.32	6	5	1
	Fukushima	Fukushima	Omori Shiroyama Park	62.8	1.13	4	3	1
			Nitanda, Omori	62.8	1.25	10	6	4
		Motomiya	Keikou Park 1	58.8	3.09	11	9	2
			Keikou Park 2*	58.8	2.73	—	—	—
		Koriyama	Furusato-no-kawa Park	60.9	2.22	3	3	0
		Hirono	Futatsunuma Park	20.7	1.30	17	12	5
		Iwaki	Shiraiwa, Yotsukura	33.3	0.46	5	3	2
			Tairaizumizaki, Buryo	37.6	0.63	19	15	4
	Ibaraki	Takahagi	Shimotetsuna	82.3	0.30	6	4	2
			Akiyama	84.1	0.42	18	15	3
		Mito	Kairakuen	127.5	0.18	4	2	2
			Mito Sports Park	130.1	0.14	10	7	3
		Tsukuba	Douhou Park 1	172.0	0.16	3	1	2
			Douhou Park 2*	172.0	0.15	—	—	—
			Sakura Sports Park 1	168.3	0.17	14	11	3
			Sakura Sports Park 2*	168.3	0.16	—	—	—
	Tokyo	Tokyo	Hibiya Park 1	225.5	0.15	14	14	0
			Hibiya Park 2*	225.5	0.09	—	—	—

* Radiation measurement only.

Table S2. Collection localities in the fall of 2011.

Collection date	Prefecture	City	Location	Distance from the NPP (km)	Ground radiation dose (μSv/h)	Number of collected		
						Total	Male	Female
20 July	Okinawa	Nishihara	Ryukyu University	1763.1	0.03**	20	8	12
18-21 September	Fukushima	Fukushima	Omori Shiroyama Park	63.1	0.71	37	31	6
			Hanamiyama	59.2	2.43	15	13	2
		Motomiya	Keikou Park 1	58.6	1.75	31	22	9
			Keikou Park 2*	58.6	1.49	—	—	—
		Hirono	Futatsunuma Park 1	21.1	0.81	19	14	5
			Futatsunuma Park 2	20.8	0.82	6	5	1
			Futatsunuma Park 3	21.3	0.90	1	1	0
		Iwaki	Shiraiwa Yotsukura	33.2	0.40	8	8	0
			Ishimoriyama	38.8	0.42	48	26	22
	Ibaraki	Kita-Ibaraki	Nakagou SA Outbound	79.8	0.24	6	2	4
		Mito	Tano SA Outbound	125.7	0.19	6	5	1
		Tsukuba	Yatabe-higashi SA Outbound	173.3	0.18	15	15	0
3-4 October	Hyogo	Kobe	Nebutani Park	596.0	0.08	46	26	20

* Radiation measurement only. ** Measured in September in the laboratory. SA: service area in the Jouban Highway.

Table S3. Collection localities in the spring of 2012.

Collection date	Prefecture	City	Location	Distance from the NPP (km)	Ground radiation dose (μSv/h)	Number of collected Total	Male	Female
12-16 May	Fukushima	Fukushima	Omori Shiroyama Park	63.2	1.12	23	19	4
			Omori, Kachuuchi	63.3	1.02	1	1	0
		Motomiya	Keikou Park 1	58.6	2.27	3	2	1
			Keikou Park 2*	58.6	2.95	–	–	–
			Janohana Yurakuen	59.0	1.47	1	0	1
		Hirono	Futatsunuma Park 1	20.6	0.87	4	2	2
			Futatsunuma Park 2*	20.4	1.03	–	–	–
			Kawarada, Shimoasamigawa	24.0	–	1	1	0
		Iwaki	Ishimori, Tairayotsunami	38.8	0.47	2	2	0
			Cyukyo-ji Temple	38.5	0.57	4	4	0
			Kaizaka, Tairashimokatayose	39.2	–	3	3	0
			Nukatsuka, Tairayotsunami	40.2	0.33	2	2	0
			Omotegawa, Tairashimokabeya	40.9	0.49	39	29	10
	Ibaraki	Takahagi	Akiyama 1	83.9	0.30	44	32	12
			Akiyama 2*	84.0	0.55	–	–	–
		Mito	Mito sports Park	130.1	0.21	2	2	0
			Kairakuen	127.6	0.18	20	18	2
			Midori bridge	127.3	0.14	0	0	0
		Tsukuba	Sakura Sports Park 1	168.3	0.17	17	14	3
			Sakura Sports Park 2*	168.2	0.21	–	–	–
	Okinawa	Nishihara	Nishihara Sports Park	1764.8	–	3	2	1
		/Urasoe	Urasoe Park 1	1765.2	0.03	11	8	3
			Urasoe Park 2*	1765.2	0.03	–	–	–

* Radiation measurement only.

Table S4. Collection localities in the fall of 2012.

Collection date	Prefecture	City	Location	Distance from the NPP (km)	Ground radiation dose (μSv/h)	Number of collected		
						Total	Male	Female
27-29 September	Aomori	Fukaura	Iriaizaki	373.8	0.04	63	39	24
			WeSPa Tsubakiyama	367.2	0.04	19	9	10
			Mozawayagishi, Hiroto	373.5	ー	3	3	0
			Koiso, Shodojiri	364.3	ー	2	0	2
			Sawabe	366.2	ー	7	5	2
			Kamihamamatsu, Matsukami	359.2	ー	1	1	0
15-20 September	Miyagi	Sendai	Chiyo bridge	90.0	0.08	76	39	36
			Rokuchounome-kita park	92.9	0.08	22	17	5
		Shiroishi	Shiroishigawa Green Park	75.0	0.23	40	29	11
	Fukushima	Fukushima	Matsukawa PA Outbound	58.7	0.84	9	6	3
			Nitanda, Omori	63.3	0.64	45	29	16
			Takinoshita, Watari	59.2	1.24	51	41	10
		Nihonmatsu	Kasumi Castle	56.6	1.06	19	16	3
		Motomiya	Keikou Park	58.8	1.29	31	26	5
			Shinsuke, Arai	57.4	1.52	12	12	0
			Janohana Yurakuen	58.6	0.61	33	27	6
		Koriyama	Furusato-no-kawa Park	60.9	1.81	43	31	12
			Araike Farming Park	58.2	(0.47)	32	15	17
		Aizuwakamatsu	Sougo Sports Park	98.1	0.47	29	20	9
		Aizumisato	Seseragigawa Park	99.4	0.15	18	13	5
		Naraha	Sougo Sports Park	15.7	0.74	1	1	0
			Mukaenouchi, Ide	15.0	0.96	1	1	0
		Hirono	Futatsunuma Park	21.1	1.29	32	22	10
			Hirono Elementary School	23.5	(0.09)	2	2	0
			Matsumoto Shrine	22.8	0.31	5	2	3
			Nagahata, Kamikitaba	23.5	0.42	20	10	10
			Nameshida, Kamikitaba	21.6	0.84	5	4	1
		Iwaki	Kairyu-no-sato Center	29.0	0.35	49	33	16
			Omotegawa, Tairashimokabeya	40.9	0.28	54	34	20
			Ishimori, Tairayotsunami	38.9	0.24	21	14	7
	Ibaraki	Takahagi	Akiyama	84.1	0.25	59	45	14
			Honmachi-minami Park	84.0	0.18	55	37	18
		Mito	Kairakuen	127.7	0.09	61	35	26
			Horihara Sports Park	125.8	0.12	62	56	6
		Tsukuba	Yatabe-higashi SA Outbound	173.3	0.18	42	27	15
			Sakura Sports Park	168.3	0.12	47	31	16
11 August	Hyogo	Kobe	Nebutani Park	596	0.07	82	52	30
25 September	Ehime	Matsuyama	Shinonome Shrine	846.3	0.12†	28	23	5
3 October	Yamaguchi	Ube	Kounan-kita	965.4	0.13†	74	61	13
24 September	Okinawa	Urasoe	Urasoe Park	1765.3	0.02	35	25	28

†Measured by DoseRAE2. Ground radiation dose after decontamination by government are indicated in parentheses.

Table S5. Collection localities in the spring of 2013.

Collection date	Prefecture	City	Location	Distance from the NPP (km)	Ground radiation dose (μSv/h)	Number of collected		
						Total	Male	Female
19-24 May	Miyagi	Sendai	Chiyo Bridge	89.9	0.11	34	28	7
		Shiroishi	Shiroishigawa Green Park	75.1	0.35	4	4	1
			Yayaguchi Fukuokakuramoto	76.7	0.23	2	2	0
			Shiroishi Castle	74.4	0.26	9	6	3
	Fukushima	Fukushima	Nitanda, Omori	63.2	0.71	19	14	5
			Takinoshita, Watari	59.3	1.73	19	16	3
		Motomiya	Kubota, Arai	58.8	1.29	5	4	1
			Janohana Park	58.7	1.12	11	7	4
			Keikou Park	58.6	1.87	0	0	0
		Koriyama	Furusato-no-kawa Park1	60.9	2.92	11	10	1
			Furusato-no-kawa Park 2*	60.9	1.18	―	―	―
			Araike Farming Park	58.3	(0.63)	1	1	0
		Hirono	Futatsunuma Park	20.6	0.67	10	7	3
			Ohira, Oriki	24.6	0.48	4	2	2
			Abuki, Kamiasamigawa	23.8	0.52	10	8	2
			Terasho, Kamiasamigawa	23.8	0.47	47	43	4
	Ibaraki	Iwaki	Kairyu-no-sato Center	28.8	0.35	8	5	3
			Omotegawa, Tairashimokabeya	40.9	0.33	46	39	7
		Takahagi	Akiyama 1	83.9	0.21	17	9	8
			Akiyama 2*	84.0	0.28	―	―	―
		Mito	Kairakuen	127.6	0.13	3	3	0
			Horihara Sports Park	125.8	0.16	1	0	1
			3 Midoricho	126.9	0.16	4	2	2
		Tsukuba	Yatabe-higashi SA Outbound	173.3	0.26	3	3	0
			Hanabatakekinrin Park	166.4	0.11	5	3	2
			Douhou Park	171.7	0.13	1	1	0
			Sakura Sports Park	168.3	0.19	0	0	0
	Fukuoka	Dazaifu	Kanzeonji	1047.2	0.08	6	5	1
		Onojo	Mikasagawa, Onojo	1048.5	―	1	1	0
	Kagoshima	Kirishima	Noguchi Bridge	1134.2	―	15	9	6
			Kokubu Sports Park	1131.2	―	4	3	1

* Radiation measurement only. Ground radiation dose after decontamination by government are indicated in parentheses.

Table S6. Collection localities in the fall of 2013.

Collection date	Prefecture	City	Location	Distance from the NPP (km)	Ground radiation dose (μSv/h)	Number of collected		
						Total	Male	Female
29, 30	Miyagi	Sendai	Chiyo Bridge	89.9	0.10	60	37	23
September			Moniwa, Taihaku	92.1	0.07	28	15	13
and 1-4		Shiroishi	Yayaguchi, Fukuokakuramoto	76.7	0.28	45	32	13
October			Shiroishigawa Green Park	75.1	0.19	32	29	3
	Fukushima	Fukushima	Nitanda, Omori	63.2	0.68	53	34	19
			Takinoshita, Watari	59.3	1.57	36	27	9
		Motomiya	Keikou Park 1	58.6	1.08	23	16	7
			Keikou Park 2*	58.8	0.85	—	—	—
			Janohana Park	58.7	1.06	37	18	19
		Koriyama	Furusato-no-kawa Park	60.9	2.20	33	23	10
			Araike Farming Park	58.3	(0.51)	16	11	5
		Hirono	Futatsunuma Park 1	20.6	0.48	25	22	3
			Futatsunuma Park 2*	20.4	0.62	—	—	—
			Terasho, Kamiasamigawa	23.8	0.39	39	26	13
		Iwaki	Kairyu-no-sato Center	28.8	0.31	27	17	10
			Omotegawa, Tairashimokabeya	40.9	0.27	50	27	23
	Ibaraki	Takahagi	Akiyama 1	83.9	0.16	48	30	18
			Akiyama 2*	84.0	0.92	—	—	—
			Honmachi-minami Park	84.0	0.17	7	7	0
		Mito	Kairakuen	127.6	0.12	27	10	17
			Horihara Sports Park	125.8	0.18	15	11	4
			3 Midoricho	126.9	0.13	19	18	1
		Tsukuba	Yatabe-higashi SA Outbound	173.3	0.18	21	21	0
			Sakura Sports Park	168.3	0.10	30	23	7
			Hanabatakekinrin Park	166.4	0.08	8	7	1
10 August	Fukushima	Fukushima	Watari	60.5	—	71	47	24
9, 11, 12, 14,		Iitate	Komiya	35.3	—	38	29	9
22 and 25-27 July			Yamatsumi Shrine	46.0	—	1	1	0
24, 25 August	Kagoshima	Kirishima	Noguchi Bridge	1134.2	0.04**	43	38	5
			Kokubu Sports Park	1131.2	—	20	12	8
		Kagoshima	Sakurajimashizenkyoryu Park	1154.8	0.03**	—	—	—

* Radiation measurement only. ** Measured in November. Ground radiation doses after decontamination by government are indicated in parentheses.
