# Supplementary figures and images for: Spatiotemporal abnormality dynamics of the pale grass blue butterfly: three years of monitoring (2011–2013) after the Fukushima nuclear accident
Source: BMC Evol Biol. 2015 Feb 10;15:15. doi: 10.1186/s12862-015-0297-1 (PMC4335452; doi:10.1186/s12862-015-0297-1)

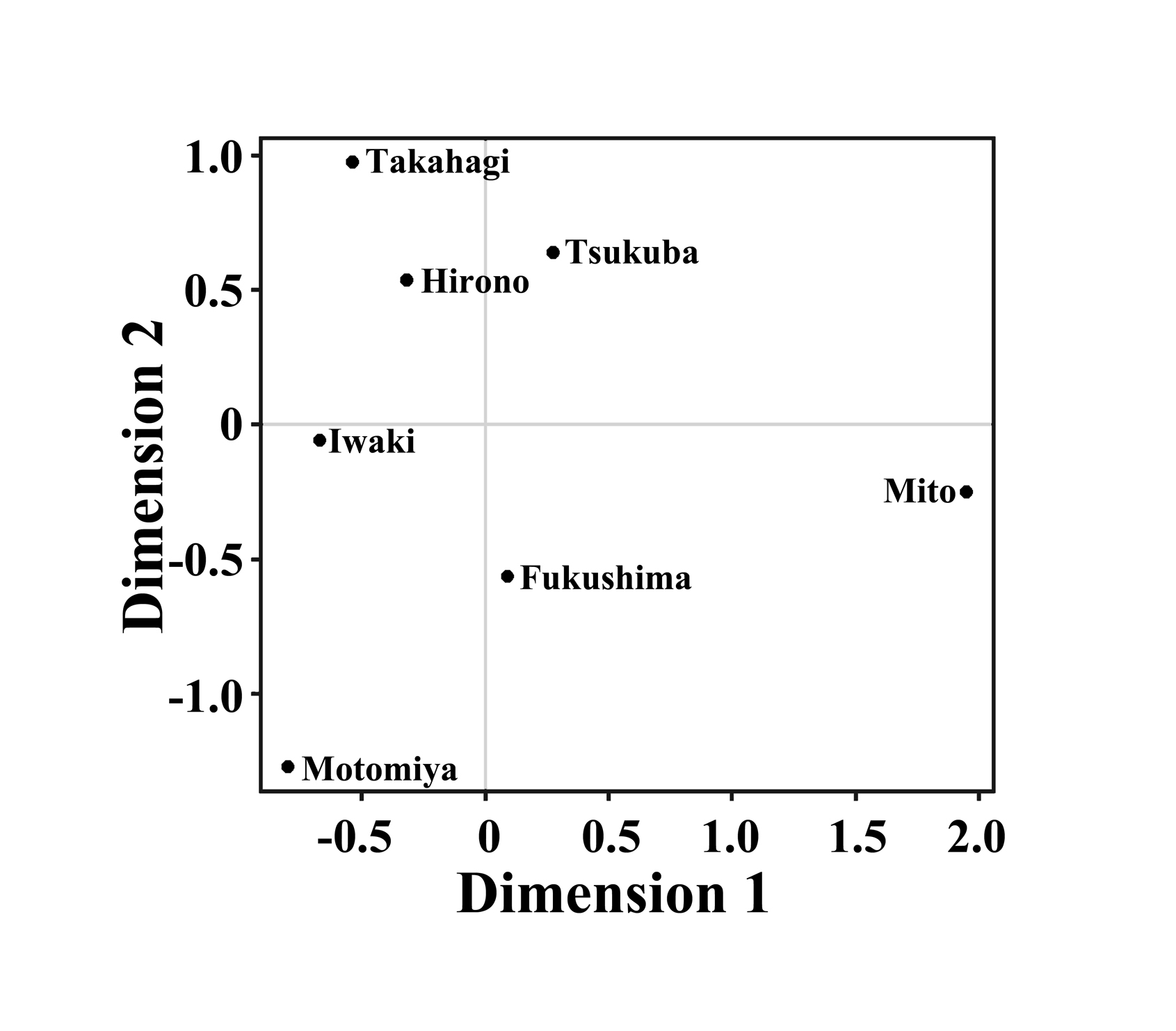

Supplement: Additional file 2: Figure S1. — A multidimensional scaling plot for the adult abnormality rate (aAR) of the P generation in 7 localities in 2011–2013. Mito is positioned far from other localities. [file 12862_2015_297_MOESM2_ESM.jpeg]

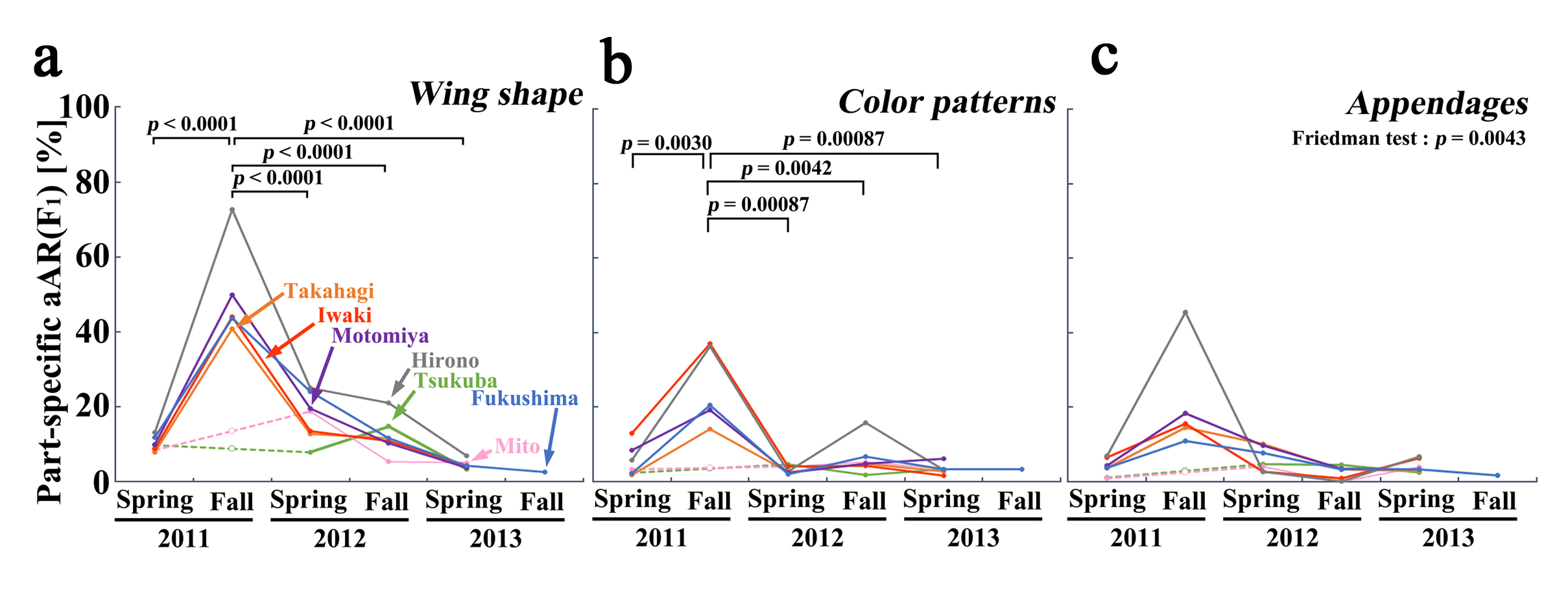

Supplement: Additional file 4: Figure S2. — Dynamics of the adult abnormality rate (AR) in the offspring (F1) generation in 2011–2013 based on different categories. (a) Wing-shape-specific aAR(F1) dynamics. (b) Color-pattern-specific AR(F1) dynamics. (c) Appendage-specific aAR(F1) dynamics. [file 12862_2015_297_MOESM4_ESM.jpeg]

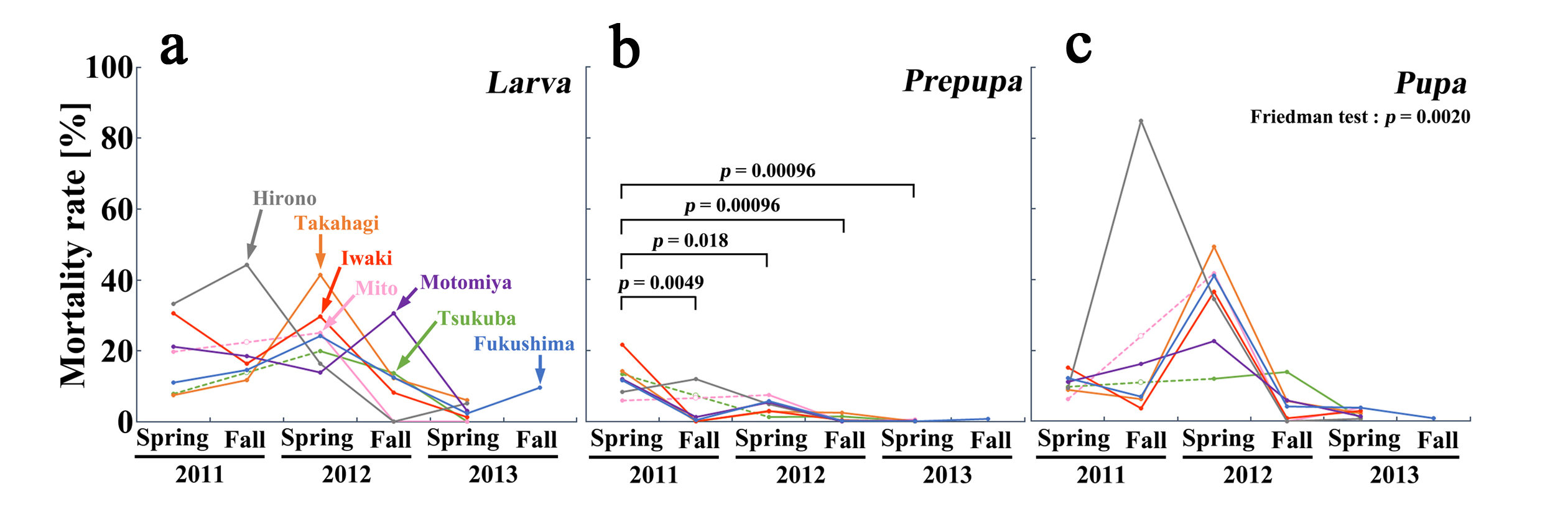

Supplement: Additional file 5: Figure S3. — Stage-specific mortality rate (MR) in 7 different localities. (a) Larval MR dynamics. Broken lines indicate lack of data in the fall of 2011 (also in the next panels). (b) Prepupal MR dynamics. (c) Pupal MR dynamics. [file 12862_2015_297_MOESM5_ESM.jpeg]

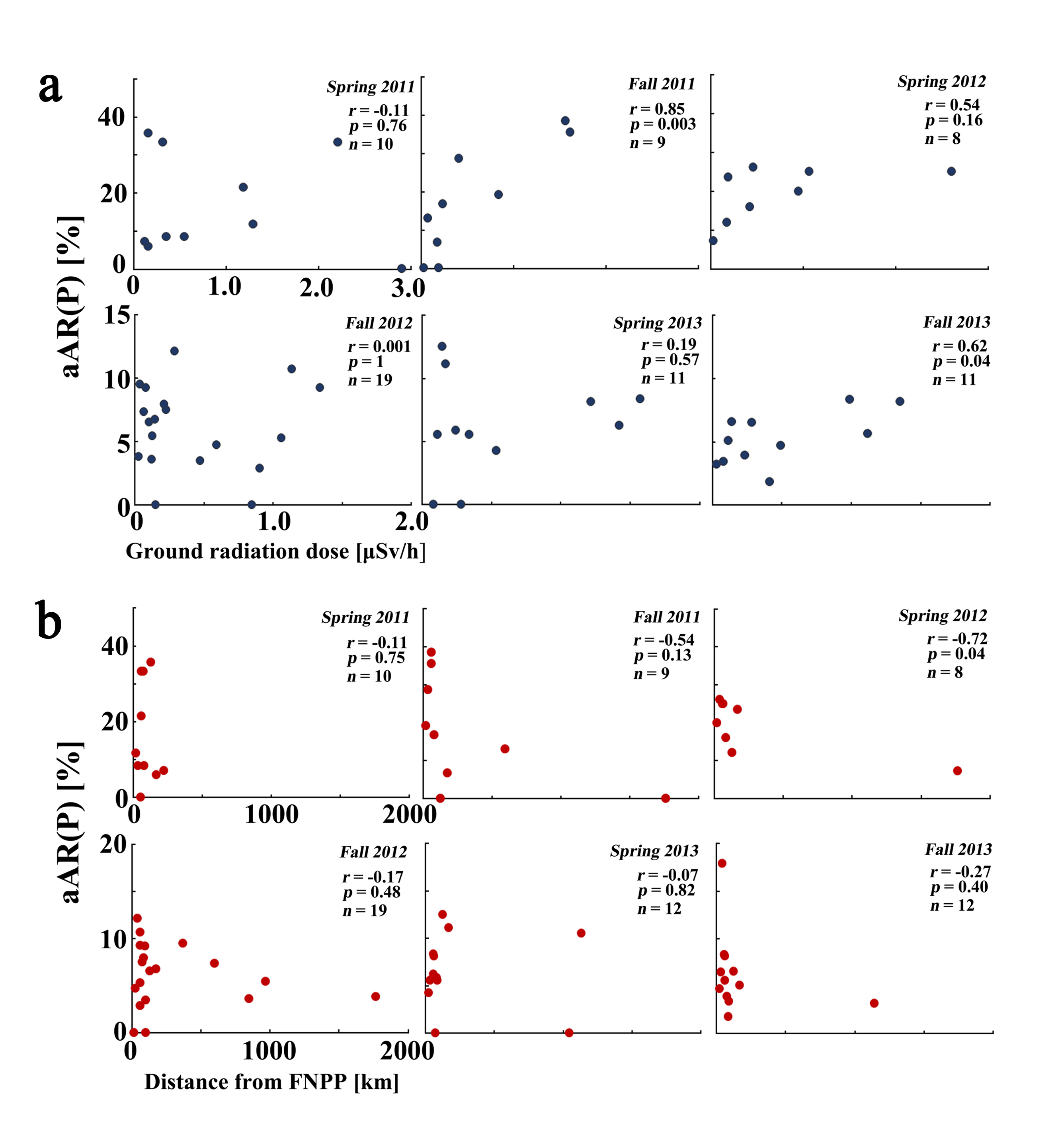

Supplement: Additional file 6: Figure S4. — Scatter plots of aAR(P). (a) Scatter plots of aAR(P) and the distance of the collection localities from the FNPP. Pearson correlation coefficients are shown together with p-values and the number of collection localities (n). (b) Scatter plots of aAR(P) and the ground radiation dose. Pearson correlation coefficients are shown together with p-values and the number of localities (n) that were subjected to radioactivity measurements. [file 12862_2015_297_MOESM6_ESM.jpeg]

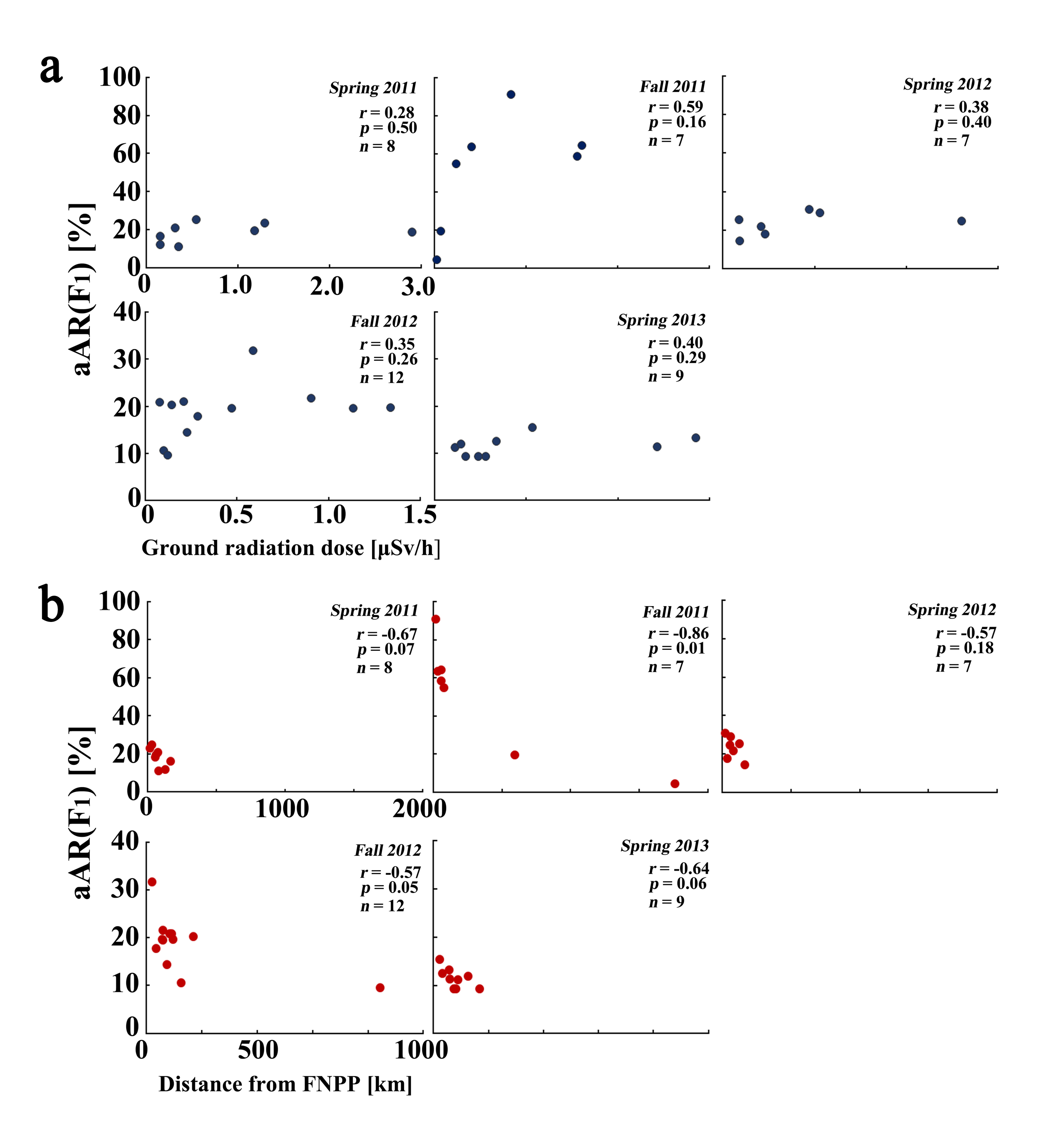

Supplement: Additional file 7: Figure S5. — Scatter plots of aAR(F1). (a) Scatter plots of aAR(F1) and the ground radiation dose. Pearson correlation coefficients are shown together with p-values and the number of localities (n) that were subjected to radioactivity measurements. (b) Scatter plots of aAR(F1) and the distance from the FNPP. Pearson correlation coefficients are shown together with p-values and the number of localities (n) that were subjected to radioactivity measurements. [file 12862_2015_297_MOESM7_ESM.jpeg]

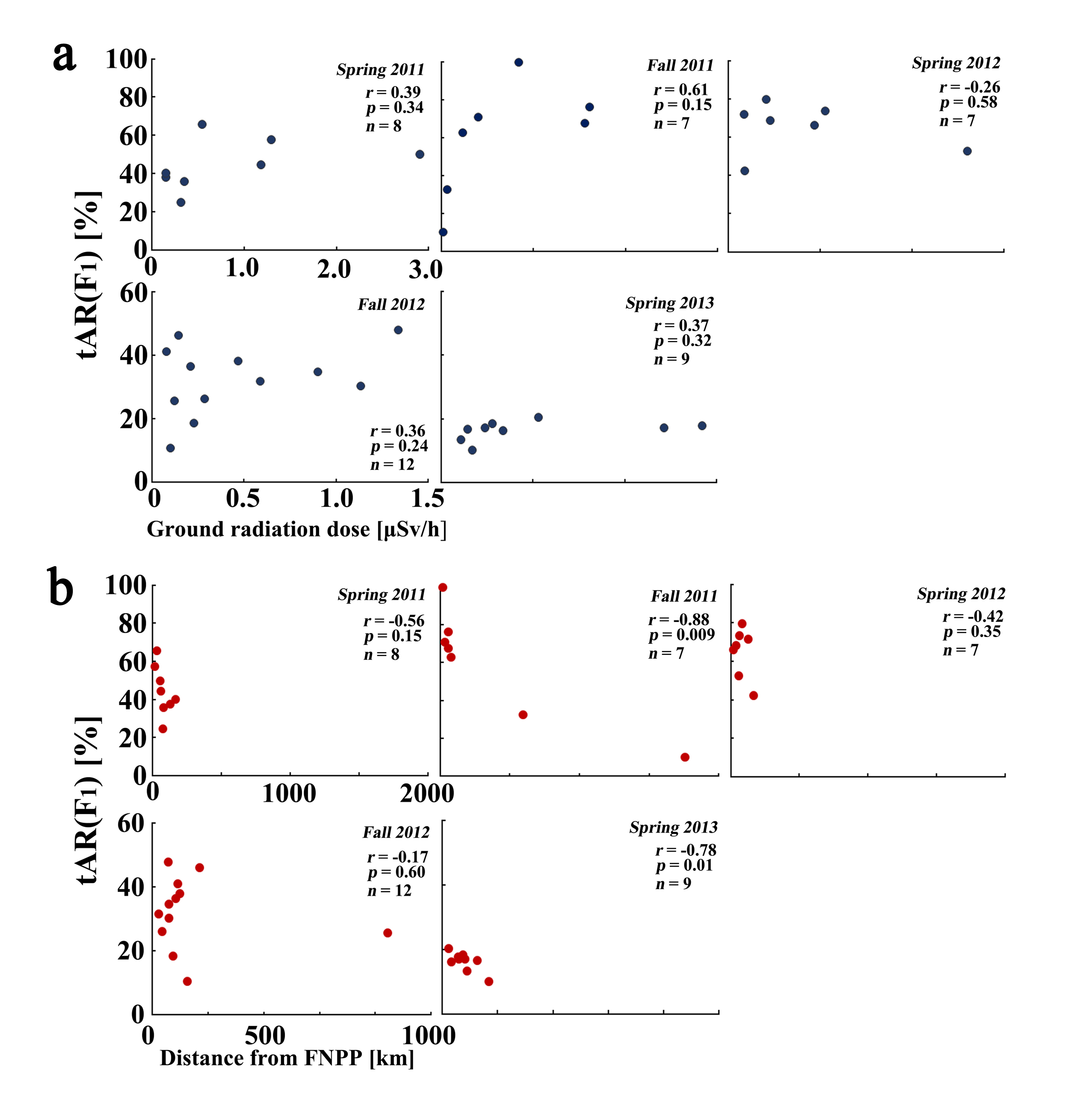

Supplement: Additional file 8: Figure S6. — Scatter plots of tAR(F1). (a) Scatter plots of tAR(F1) and the ground radiation dose. Pearson correlation coefficients are shown together with p-values and the number of localities (n) that were subjected to radioactivity measurements. (b) Scatter plots of tAR(F1) and the distance from the FNPP. Pearson correlation coefficients are shown together with p-values and the number of localities (n) that were subjected to radioactivity measurements. [file 12862_2015_297_MOESM8_ESM.jpeg]
