# Supplementary material for: Spatiotemporal abnormality dynamics of the pale grass blue butterfly: three years of monitoring (2011–2013) after the Fukushima nuclear accident
Source: BMC Evol Biol. 2015 Feb 10;15:15. doi: 10.1186/s12862-015-0297-1 (PMC4335452; doi:10.1186/s12862-015-0297-1)
Supplement: Additional file 3: Table S7. — Number of adults used for the egg collection process. [file 12862_2015_297_MOESM3_ESM.pdf]

Table S7. Number of adults used for the egg collection process.

	/ Year	Number of adults used (*n* (Female)) 2011		2012		2013	
Prefecture	City / Season	Spring	Fall	Spring	Fall	Spring	Fall
Miyagi	Sendai	—	—	—	12 (8)	9 (6)	—
	Shiroishi	3 (1)	—	—	9 (6)	6 (4)	—
Fukushima	Fukushima	8 (5)	9 (5)	7 (4)	22 (14)	9 (6)	8 (7)
	Motomiya	5 (2)	7 (5)	4 (2)	12 (7)	8 (5)	—
	Koriyama	—	—	—	10 (6)	—	—
	Aizuwakamatsu	—	—	—	10 (6)	—	—
	Hirono	7 (4)	5 (3)	5 (2)	9 (5)	9 (6)	—
	Iwaki	8 (6)	6 (4)	6 (3)	13 (9)	9 (6)	—
Ibaraki	Takahagi / Kita-Ibaraki	7 (4)	6 (4)	6 (3)	10 (6)	9 (6)	—
	Mito	9 (5)	—	6 (3)	8 (5)	4 (2)	—
	Tsukuba	8 (5)	—	6 (3)	15 (10)	4 (2)	—
Hyogo	Kobe	—	7 (5)	—	—	—	—
Ehime	Matsuyama	—	—	—	7 (4)	—	—
Okinawa	Nishihara/Urasoe	—	20 (12)	—	—	—	—
